# The chromatin remodeling factor BAP18 promotes non–small cell lung cancer progression *via* the recruitment of β-catenin with the transcriptional coactivator complex ACTL6A–PAF1

**DOI:** 10.1016/j.jbc.2025.110596

**Published:** 2025-08-14

**Authors:** Junli Hao, Qilin Hu, Xin Li, Sha Shi, Fangjian Na, Kai Zeng, Hao Li, Yue Zhao, Mingfang Zhao

**Affiliations:** 1 Department of Medical Oncology, The First Hospital of China Medical University, Shenyang, Liaoning, China; 2 Network Information Center, China Medical University, Shenyang, Liaoning, China; 3 Department of Cell Biology, Key Laboratory of Medical Cell Biology, Ministry of Education, School of Life Sciences, China Medical University, Shenyang, Liaoning, China

**Keywords:** non–small cell lung cancer, BAP18, β-catenin, transcriptional regulation, epigenetics

## Abstract

Non–small cell lung cancer (NSCLC) is a prevalent and deadly form of lung cancer, with treatment challenges including drug resistance and limited therapeutic targets, despite advances, such as immune checkpoint inhibitors. This study investigated the role of BAP18 (BPTF-associated protein of 18 kDa), a chromatin-associated protein, in NSCLC progression and its potential as a therapeutic target. NSCLC tissue samples were analyzed for BAP18 expression using Western blot and immunohistochemistry, and NSCLC cell lines with BAP18 knockdown were assessed for proliferation, migration, cell cycle, and tumor growth through *in vitro* assays and xenograft models. Coimmunoprecipitation and luciferase reporter assays were used to explore the interaction of BAP18 with β-catenin, ACTL6A (actin like 6A), and PAF1 (polymerase-associated factor 1) and its impact on β-catenin-mediated transcriptional activity. RNA sequencing and enrichment analyses identified the pathways involved in BAP18-regulated NSCLC progression. The results showed that BAP18 is highly expressed in NSCLC tissues, and its knockdown significantly inhibited cell proliferation, migration, and tumor growth. Mechanistically, BAP18 recruits ACTL6A and PAF1 to Wnt (wingless/integrated) target gene promoters, enhancing β-catenin-mediated transcription. These findings suggest that BAP18 plays a critical role in NSCLC progression through the Wnt–β-catenin pathway and could serve as a novel therapeutic target, particularly for patients with Wnt–β-catenin-driven tumors.

Lung cancer is a highly prevalent and lethal malignant tumor, classified into small cell lung cancer and non–small cell lung cancer (NSCLC), with NSCLC accounting for 80% of cases (1). According to the recent US cancer statistics, lung cancer ranks second in new cancer cases, causing over 350 deaths daily, surpassing the combined deaths from breast, prostate, and pancreatic cancers (2). Despite increased public awareness of early diagnosis and screening, most patients are diagnosed at an advanced stage (3). Prior to the 21^st^ century, platinum-based chemotherapy was the primary treatment for advanced lung cancer and remains a fundamental component of current therapeutic strategies, often used alone or in combination with other modalities. The development of tyrosine kinase inhibitors and immune checkpoint inhibitors in recent decades has significantly advanced NSCLC treatment (4, 5). These therapies have extended survival in selected patients with locally advanced or metastatic disease, with 5-year survival rates exceeding 20% in eligible populations (6). However, the stringent selection criteria for targeted and immunotherapies and the issue of drug resistance can lead to treatment failure (7, 8). Thus, identifying new therapeutic targets remains a key focus in lung cancer research.

Epigenetic regulation involves heritable DNA modifications that change the three-dimensional arrangement of nucleosomes, thereby regulating gene expression without altering the nucleotide sequence. This includes DNA methylation, chromatin remodeling, histone modification, and noncoding RNA (9, 10, 11, 12, 13). These changes are inducible and reversible, potentially influenced by environmental factors, such as air pollution, smoking, ultraviolet radiation, and diet (14, 15, 16). Common targets for epigenetic therapy include DNA methylation and hydroxymethylation and histone modifications, such as methylation, acetylation, phosphorylation, and succinylation (17, 18, 19). These modifications can affect oncogenes and tumor suppressors, leading to aberrant molecular signaling pathways (20). Epigenetic modifications of histones allow chromatin to switch between closed and open states. Open chromatin states permit transcriptional activators to access target genes and promote transcription, whereas closed chromatin states are typically associated with transcriptional silencing (21). Modifications at different histone sites are crucial for transcriptional regulation in tumor cells; for instance, H3K4me3 and H3K36me3 are associated with transcriptional activation, whereas H3K9me3 and H3K27me3 are linked to transcriptional repression (22, 23).

Regulation at the transcriptional level involves various coregulatory factors. For example, aberrant expression of these coregulators is a key factor in the Wnt (wingless/integrated)–β-catenin signaling pathway–mediated gene transcription anomalies. The C-terminal region of β-catenin recruits numerous factors, including HATs CBP, p300, and TIP60, SWI–SNF factors BRG1, ISWI, and mediator component MED12. Many transcription factors activate transcription by sequentially or cyclically recruiting similar coregulatory factors (24, 25).

BAP18 (BPTF-associated protein of 18 kDa), encoded by the human C17orf49 gene, is a component of chromatin complexes MLL1-WDR5 (26) and NURF–BPTF (27). It contains an SANT domain, typically found in chromatin remodeling complexes. Studies show that BAP18 acts as an androgen receptor (AR) coregulator in prostate cancer, upregulating AR-mediated gene transcription and promoting prostate cancer progression (28); in estrogen receptor (ER)–positive breast cancer, BAP18 associates with the COMPASS complex, upregulating ER-mediated gene transcription, promoting ERα-positive breast cancer growth, and contributing to tamoxifen resistance (29). A recent study reported that BAP18 is upregulated in non–small cell lung carcinoma (NSCLC), particularly in advanced-stage tumors, and promotes tumor cell proliferation by enhancing the transcription of cell cycle–related genes, such as CCND1 (cyclin D1) and CCND2 (30). However, the underlying molecular mechanisms remain incompletely understood. In this study, we further explored the functional role of BAP18 in NSCLC, with a particular focus on its involvement in transcriptional regulation and tumor progression.

In this study, we found that BAP18 expression was significantly elevated in NSCLC samples. In NSCLC cell lines, BAP18 depletion led to an increased proportion of cells in the G1 phase, a decreased proportion in the S phase, slowed cell proliferation, and inhibited tumor cell growth and migration *in vitro* and *in vivo*. We further confirmed that BAP18 recruited ACTL6A (actin like 6A) and PAF1 (polymerase-associated factor 1), along with β-catenin, to the promoter region of target genes to activate the Wnt signaling pathway, leading to tumor proliferation and metastasis. These findings provide novel insights into the mechanism of the Wnt–β–catenin pathway and enhance our understanding of the role of BAP18 in NSCLC, which may have significant implications for potential therapeutic strategies.

## Results

### BAP18 is highly expressed in NSCLC and correlates with tumor malignancy

**Figure 1:**
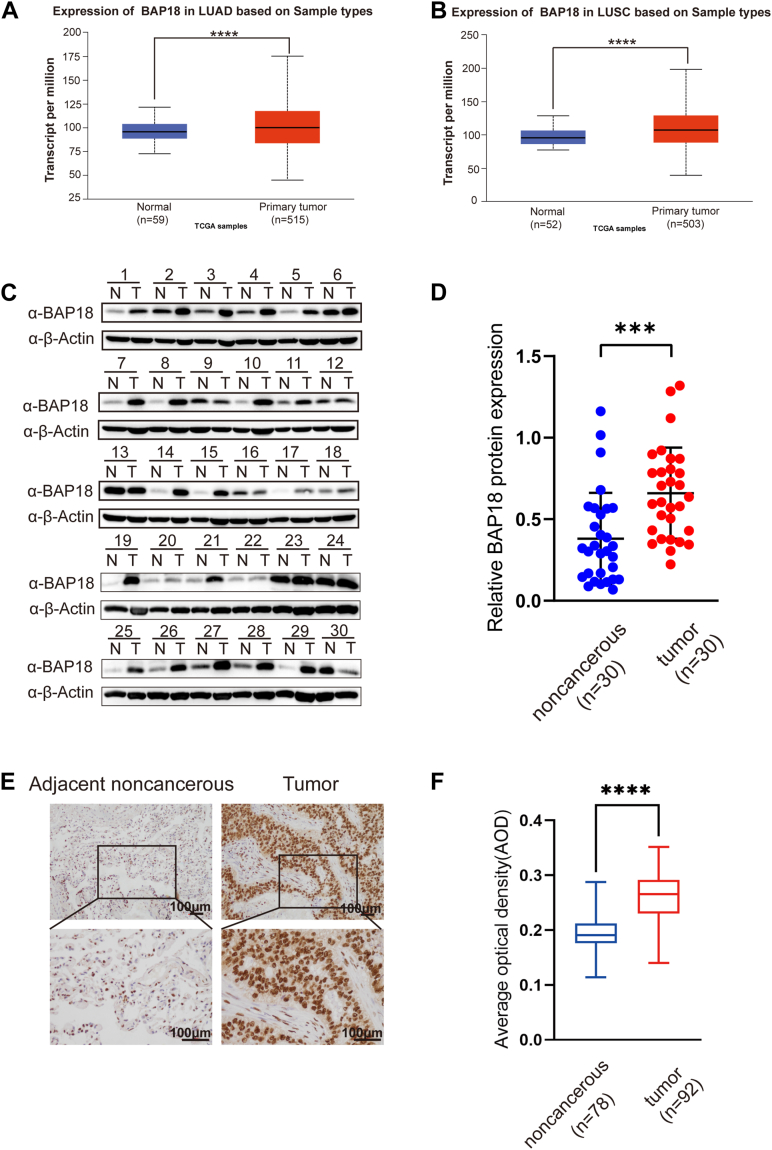
**BAP18 is upregulated in lung cancer tissues.***A* and *B*, box plots showing BAP18 mRNA expression levels in LUAD and LUSC tumor tissues compared with normal tissues, based on TCGA datasets accessed *via* the UALCAN portal (LUAD: n = 59 normal, 515 tumor; LUSC: n = 52 normal, 503 tumor). Statistical analysis was performed using unpaired two-tailed Student’s *t* test. *C* and *D*, representative Western blot analysis of BAP18 protein expression in 30 paired fresh lung cancer (T) and adjacent noncancerous (N) tissues. β-Actin served as a loading control. Data are presented as mean ± SEM. Statistical comparison was conducted using paired two-tailed Student’s *t* test. *E* and *F*, immunohistochemical staining of BAP18 in paraffin-embedded lung tumor and adjacent noncancerous tissues. Statistical analysis was performed using unpaired two-tailed Student’s *t* test. Tumor (n = 92), noncancerous (n = 78). Original blots are shown in Fig. S3. ∗∗∗∗*p* < 0.0001; ∗∗∗*p* < 0.001. BAP18, BPTF-associated protein of 18 kDa; LUAD, lung adenocarcinoma; LUSC, lung squamous cell carcinoma; TCGA, The Cancer Genome Atlas.

We initially analyzed the expression of BAP18 in tumor and normal tissues using the UALCAN online dataset (31). The results demonstrated that BAP18 expression was elevated in NSCLC tissues, including lung adenocarcinoma (LUAD) and lung squamous cell carcinoma, compared with normal tissues (Fig. 1, *A* and *B*). To confirm the higher BAP18 expression, we collected fresh NSCLC tissue samples and paraffin-embedded tissue microarrays to explore BAP18 expression in clinical specimens. We selected 30 NSCLC tissues and their paired adjacent noncancerous tissues from the First Affiliated Hospital of China Medical University and measured BAP18 protein levels using Western blot. The results showed significantly higher BAP18 expression in tumor tissues compared with adjacent normal tissues (Fig. 1, *C* and *D*). Immunohistochemical staining of tissue microarrays further confirmed that BAP18 was highly expressed in NSCLC tissues compared with adjacent normal tissues (Fig. 1, *E* and *F*). Statistical analysis of tissue microarrays showed increased BAP18 levels in patients with lymph node metastasis and higher clinical stages, suggesting a correlation between high BAP18 expression and tumor malignancy (Table 1). However, analysis of The Cancer Genome Atlas (TCGA)-LUAD database did not reveal a significant difference in BAP18 expression between classic NSCLC driver gene mutation statuses (*p* > 0.05) (Fig. S1*A*).

**Table 1:** Association between BAP18 expression and clinicopathological characteristics of NSCLC

Clinical pathological factors	Cases (n = 92)	Low BAP18 expression (n = 39)	High BAP18 expression (n = 53)	*p*
Gender				0.684
Male	52	23	29	
Female	40	16	24	
Age				0.691
<55	24	11	13	
≥55	68	28	40	
Pathological grade				0.554
Ⅰ	4	1	3	
Ⅱ	56	26	30	
Ⅲ	32	12	20	
T				0.632
T1	34	17	17	
T2	36	14	22	
T3	13	4	9	
T4	9	4	5	
N				0.024^a^
N0	44	24	20	
N1–N3	48	15	33	
Clinical stage				0.030^a^
Ⅰ	31	18	13	
Ⅱ–Ⅲ	61	21	40	
Epidermal growth factor receptor				0.434
Mutated	9	2	7	
Nonmutated	61	27	34	
Unknown	22	10	12	

Associations between BAP18 expression and clinical variables including gender, age, pathological grade, tumor (T) stage, nodal (N) stage, clinical stage, and epidermal growth factor receptor mutation status were analyzed using the Chi-square test or Fisher’s exact test as appropriate.

a *p* < 0.05 was considered statistically significant.

We also performed single sample gene set enrichment analysis on 32,368 tumor epithelial cells from the single-cell dataset GSE131907 based on BAP18 expression status. As shown in Fig. S1*B*, 1516 epithelial cells (4.68%) exhibited positive expression of BAP18. The results showed that BAP18-positive cells exhibited increased expression in proliferation and cell cycle checkpoint–related pathways (Fig. S1*C*).

### BAP18 knockdown inhibits NSCLC growth and invasion *in vitro*

**Figure 2:**
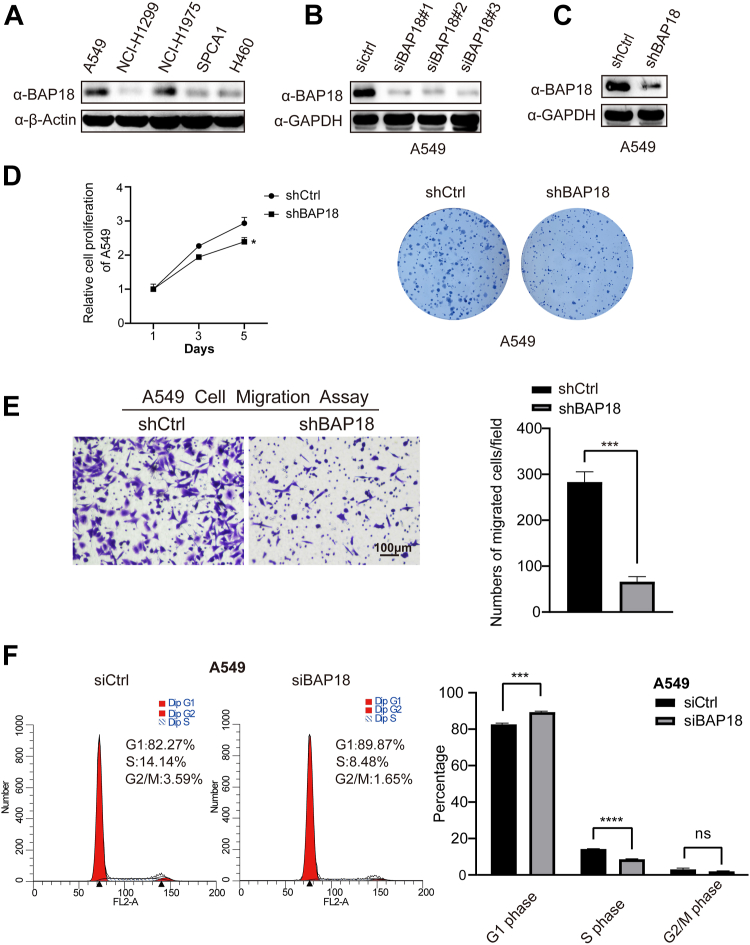
**Knockdown of BAP18 inhibits proliferation, migration, and alters cell cycle distribution in NSCLC cells.***A*, Western blot analysis of endogenous BAP18 protein expression in NSCLC cell lines. β-Actin was used as a loading control. *B*, efficiency of BAP18 knockdown in A549 cells by three siRNA constructs, assessed by Western blot. GAPDH served as a loading control. *C*, Western blot validation of lentivirus-mediated BAP18 knockdown in A549 cells. *D*, cell proliferation was assessed by CCK-8 assay over 5 days, and colony formation was evaluated after stable knockdown. *E*, transwell migration assay showing decreased migratory capacity of A549 cells upon BAP18 knockdown. Representative images (*left*) and quantification of migrated cells per field (*right*). *F*, flow cytometry analysis of cell cycle distribution in siCtrl and siBAP18-treated A549 cells. Bar plots show the percentage of cells in G1, S, and G2/M phases. Original blots are shown in Fig. S4. Data represent mean ± SEM from three independent experiments. Statistical analysis was performed using unpaired two-tailed Student’s *t* test. ∗∗∗∗*p* < 0.0001; ∗∗∗*p* < 0.001; ∗∗*p* < 0.01; ∗*p* < 0.05; and ns, no statistical significance. BAP18, BPTF-associated protein of 18 kDa; CCK-8, Cell Counting Kit-8; NSCLC, non–small cell lung cancer.

Clinical samples and database analyses suggested that BAP18 promotes NSCLC proliferation. To elucidate the biological function of BAP18 in NSCLC, we first examined BAP18 expression in NSCLC cell lines using Western blot and selected A549 and NCI-H1299 cell lines for further experiments (Fig. 2*A*). We conducted BAP18 knockdown experiments using three specific siRNAs targeting BAP18 and generated BAP18 knockdown lentivirus (shBAP18) with 1#siRNA, constructing stable BAP18 knockdown cell lines A549 and H1299 (Figs. 2, *B*, *C* and S2, *A*, *B*). Methylthiazol tetrazolium salt assays and colony formation experiments showed that BAP18 knockdown inhibited cell proliferation and reduced colony formation (Figs. 2*D* and S2*C*). Transwell and wound healing assays demonstrated that BAP18 knockdown significantly inhibited A549 cell migration (Figs. 2*E* and S2*D*).

Having observed that BAP18 promotes NSCLC cell growth and migration, we next performed cell cycle analysis to explore whether BAP18 might influence tumor cell proliferation through cell cycle regulation. BAP18 was knocked down in A549 and H1299 cells using siRNA, followed by flow cytometric analysis. The results showed an increased proportion of cells in the G1 phase and a decreased proportion in the S phase in the BAP18 knockdown group compared with controls, with no significant change in the G2–M phase (Figs. 2*F* and S2*E*). These findings suggest that BAP18 may influence cell cycle progression at the G1–S transition in NSCLC cells. However, we acknowledge that further mechanistic studies are needed to determine whether this effect contributes directly to the tumor-promoting role of BAP18 *in vivo*.

### BAP18 knockdown suppresses NSCLC tumor growth *in vivo*

**Figure 3:**
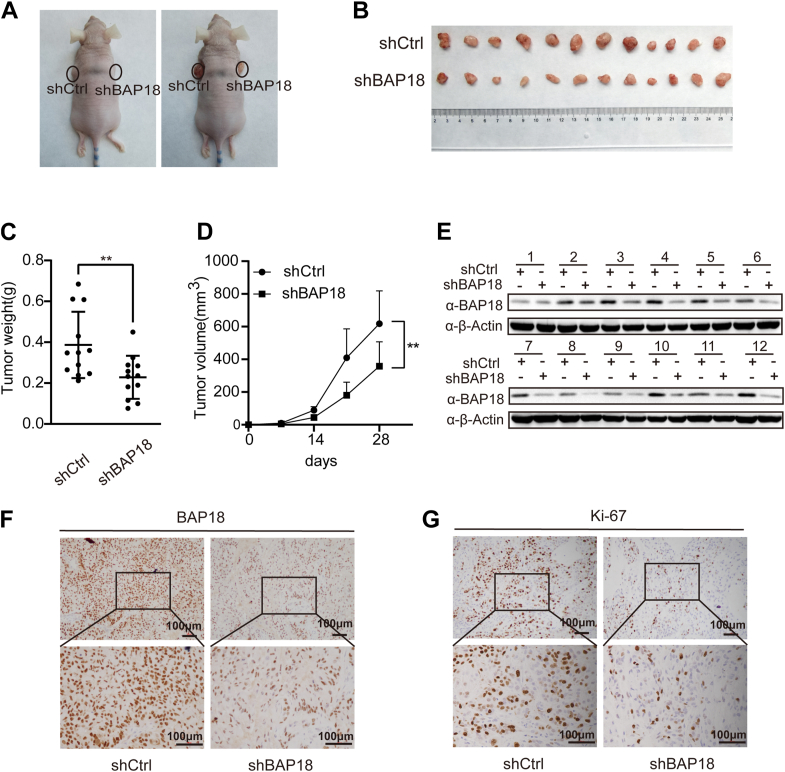
**BAP18 knockdown suppresses tumor growth in NSCLC xenograft mouse models.***A*, representative image of nude mice 4 weeks after subcutaneous injection of A549 cells stably expressing shCtrl or shBAP18. Tumors formed in the *left* (shCtrl) and *right* (shBAP18) axilla of each mouse. *B*, gross appearance of excised tumors from mice injected with shCtrl or shBAP18 A549 cells. *C*, tumor weights measured at the endpoint (day 28) for each group. *D*, tumor growth curve showing volume measurements taken on days 0, 14, and 28. *E*, Western blot analysis of BAP18 protein expression in tumor tissues from xenograft mice. β-Actin served as the loading control. *F* and *G*, representative immunohistochemical staining for BAP18 (*F*) and Ki-67 (*G*) in tumor tissues from each group. Original Western blot images are shown in Fig. S5. Data represent mean ± SEM (n = 12). Statistical analysis was performed using unpaired two-tailed Student’s *t* test. ∗∗*p* < 0.01. BAP18, BPTF-associated protein of 18 kDa; NSCLC, non–small cell lung cancer.

Our findings from experiments *in vitro* indicate that BAP18 enhances NSCLC cell proliferation and migration. To further evaluate the effect of BAP18 on tumor growth *in vivo*, we established xenograft mouse models with the NSCLC cell line A549 infected with shBAP18 or negative control lentivirus (shCtrl). After 4 weeks, tumors were excised, and their volumes and weights were measured. The results showed that tumors in the BAP18 knockdown group were smaller and lighter than those in the control group (Fig. 3, *A*–*D*). Western blot analysis of tumor tissues revealed significantly reduced BAP18 expression in the BAP18 knockdown group (Fig. 3*E*). Immunohistochemical staining showed decreased BAP18 and the proliferation marker Ki-67 in tumors. These results indicate that BAP18 knockdown can inhibit NSCLC tumor growth *in vivo*, suggesting that reduced BAP18 expression slows tumor growth (Fig. 3, *F* and *G*).

### Target genes regulated by BAP18 are related to β-catenin nuclear translocation

**Figure 4:**
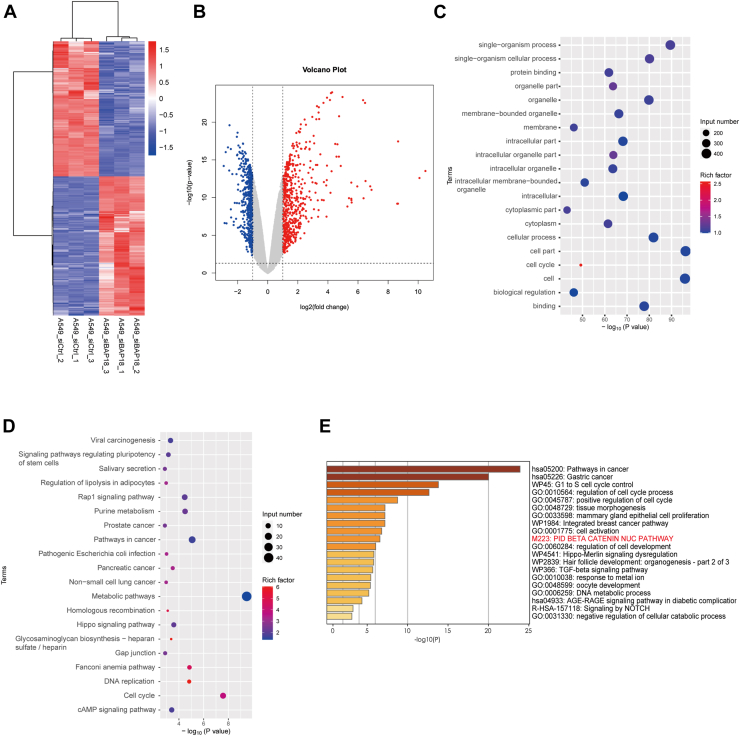
**Transcriptomic profiling identifies BAP18-associated gene expression changes and enriched signaling pathways.***A*, heatmap showing differentially expressed genes (DEGs) between BAP18 knockdown and control groups. Each *column* represents an individual sample, and *rows* represent normalized gene expression values (*Z*-score). *B*, volcano plot showing significantly upregulated (*red*, n = 580) and downregulated (*blue*, n = 563) genes following BAP18 knockdown. DEGs were defined as |log_2_(fold change)|>1 and adjusted *p* < 0.05. *C*, Gene Ontology (GO) enrichment analysis of downregulated genes after BAP18 knockdown, categorized under biological process terms. Dot size indicates the number of input genes; color denotes rich factor. *D*, KEGG pathway enrichment analysis based on DEGs affected by BAP18 knockdown. *E*, selected enrichment terms related to cell proliferation, tumorigenesis, and cell cycle regulation. Pathways include both GO and KEGG categories, and pathways are ranked by statistical significance. BAP18, BPTF-associated protein of 18 kDa; KEGG, Kyoto Encyclopedia of Genes and Genomes.

To further explore the mechanisms by which BAP18 promotes NSCLC progression, we performed transcriptomic sequencing on A549 cells in BAP18 knockdown (siBAP18) and control (siCtrl) groups, analyzing the differences across the genome. Knockdown of BAP18 resulted in significant changes in 1143 genes (*p* < 0.05, |log_2(_fold change)|>1), with 563 positively regulated differentially expressed genes (DEGs) significantly downregulated (Fig. 4, *A* and *B*). Gene Ontology (GO) functional analysis of these DEGs showed that they are involved in various functions and pathways, including single-organism processes, cellular processes, protein binding, cell cycle, and biological regulation (Fig. 4*C*). Kyoto Encylopedia of Genes and Genomes enrichment analysis indicated involvement in stem cell pluripotency, Rap1 signaling pathway, cancer, NSCLC, metabolism, Hippo signaling pathway, DNA replication, and cell cycle (Fig. 4*D*). Further analysis of a subset of genes significantly regulated by BAP18 and involved in cell growth and tumor progression revealed that these genes affect tumor development through cell cycle progression, transforming growth factor-β signaling pathway, and β-catenin nuclear translocation signaling pathway (Fig. 4*E*). β-catenin is a crucial transcriptional coactivator in the Wnt signaling pathway, regulating Wnt target gene expression. In the absence of Wnt signaling, β-catenin is sequestered in the cytoplasm by a destruction complex composed of APC, AXIN, CK1, and GSK3β and degraded by the proteasome upon phosphorylation. When Wnt signaling is present, β-catenin translocates into the nucleus, binds to T-cell factor (TCF)–lymphoid enhancer–binding factor (LEF), and activates transcription of Wnt target genes.

Transcriptomic sequencing results indicate that BAP18 knockdown alters the expression of several genes, including components of the Wnt signaling pathway, in NSCLC cell lines.

### BAP18 upregulates β-catenin-mediated transcriptional activity and promotes downstream target gene expression

**Figure 5:**
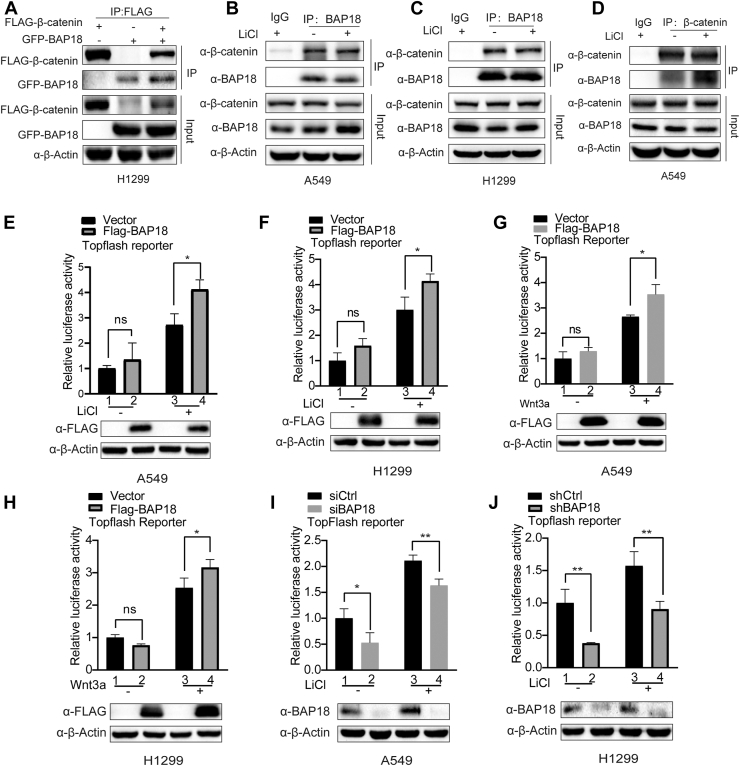
**BAP18 interacts with β-catenin and upregulates β-catenin-mediated gene transcription.***A*, coimmunoprecipitation (Co-IP) assay showing exogenous interaction between FLAG-tagged β-catenin and GFP-tagged BAP18 in H1299 cells. Lysates were immunoprecipitated with anti-FLAG and probed for BAP18 and β-catenin. *B*–*D*, endogenous interaction between BAP18 and β-catenin in A549 and H1299 cells with or without LiCl stimulation (20 mM, 24 h). *E*–*H*, luciferase reporter assays using the TopFlash reporter to assess β-catenin transcriptional activity in A549 and H1299 cells transfected with FLAG-BAP18 or vector control, treated with LiCl (20 mM) or Wnt3a (50 ng/ml) for 24 h. Luciferase activity was normalized to protein levels. *I* and *J*, luciferase reporter assays in A549 (*I*) and H1299 (*J*) cells after BAP18 knockdown using siRNA or shRNA, respectively, with or without LiCl stimulation. β-Actin was used as a loading control. Original blots are shown in Fig. S6. Data are shown as mean ± SEM from three independent experiments. Statistical analysis was performed using unpaired two-tailed Student’s *t* test. ∗∗*p* < 0.01; ∗*p* < 0.05; ns, no statistical significance. BAP18, BPTF-associated protein of 18 kDa; LiCl, lithium chloride.

We conducted exogenous and endogenous co-IP experiments in A549 and H1299 cells to investigate the relationship between BAP18 and β-catenin. The results showed that exogenously transfected BAP18 and β-catenin could interact, and endogenous BAP18 and β-catenin interacted in cells (Fig. 5, *A*–*D*). As BAP18 is a coregulator, it remained unclear whether it participates in β-catenin-mediated Wnt signaling pathway transcriptional activation. Therefore, we conducted dual-luciferase reporter assays. The results demonstrated that BAP18 upregulated β-catenin-mediated gene transcriptional activity in the presence of lithium chloride (LiCl) or Wnt3a recombinant protein stimulation (Fig. 5, *E*–*H*). Subsequent dual-luciferase reporter assays in BAP18 knockdown A549 and H1299 cells showed that silencing BAP18 reduced β-catenin-mediated gene transcriptional activity (Fig. 5, *I* and *J*).

**Figure 6:**
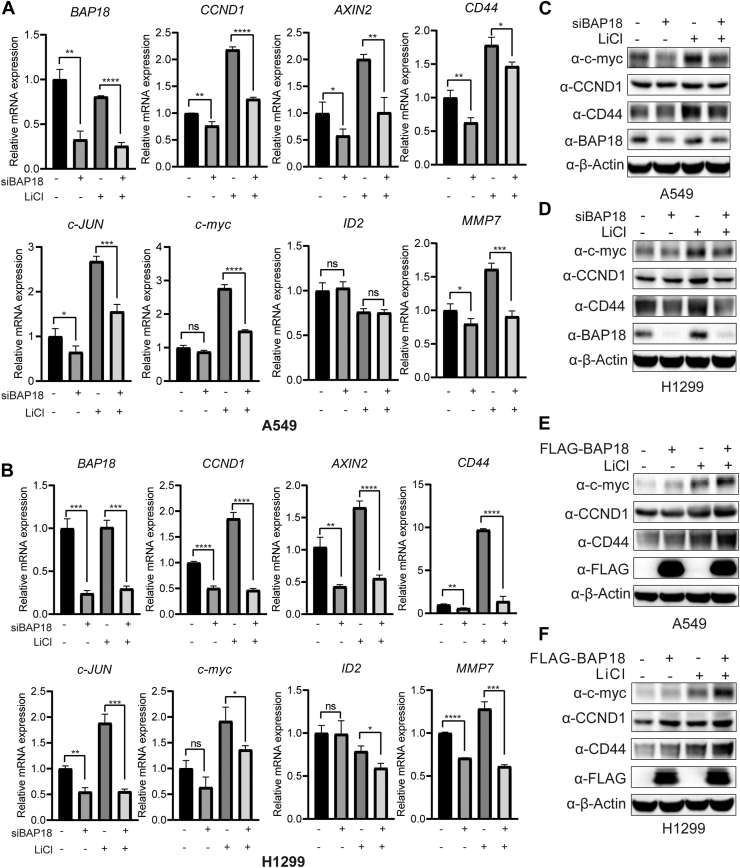
**BAP18 modulates the transcription and protein expression of β-catenin downstream target genes.***A* and *B*, quantitative real-time PCR analysis of mRNA expression levels of β-catenin target genes (cyclin D1 [CCND1], AXIN2, CD44, c-JUN, c-MYC, ID2, and MMP7) in A549 (*A*) and H1299 (*B*) cells following BAP18 knockdown by siRNA (siBAP18) or control siRNA (siCtrl), with or without LiCl stimulation (20 mM, 12 h). *C* and *D*, Western blot analysis of c-Myc, CCND1, and CD44 protein levels in A549 (*C*) and H1299 (*D*) cells following BAP18 knockdown, with or without LiCl stimulation. β-Actin was used as a loading control. *E* and *F*, Western blot analysis of c-Myc, CCND1, and CD44 protein levels in A549 (*E*) and H1299 (*F*) cells transfected with FLAG-BAP18 or control vector, treated with or without LiCl. Original blots are provided in Fig. S7. Data are shown as mean ± SEM from three independent experiments. Statistical analysis was performed using unpaired two-tailed Student’s *t* test. ∗∗∗∗*p* < 0.0001; ∗∗∗*p* < 0.001; ∗∗*p* < 0.01; ∗*p* < 0.05; ns, no statistical significance. BAP18, BPTF-associated protein of 18 kDa; LiCl, lithium chloride.

To clarify the effect of BAP18 on β-catenin-mediated Wnt signaling pathway target gene expression, we selected classical Wnt pathway target genes for RNA and protein level validation. BAP18 expression was knocked down in A549 and NCI-H1299 cells using siRNA, with siCtrl as the control group, and 20 mM LiCl was added for stimulation, with NaCl as the negative control. After 12 h of treatment, RNA was collected for quantitative PCR (qPCR) analysis. The results showed that siRNA significantly reduced BAP18 mRNA levels, and under LiCl stimulation, CCND1, AXIN2, CD44, c-JUN, c-myc, and matrix metalloproteinase 7 (MMP7) mRNA levels were significantly increased compared with the control group (Fig. 6, *A* and *B*). Knockdown of BAP18 resulted in decreased levels of these mRNAs, whereas ID2 mRNA levels were not significantly affected. After 24 h of treatment, protein was extracted for Western blot analysis. Under LiCl stimulation, CCND1, c-myc, and CD44 protein levels increased, and BAP18 knockdown reduced these protein levels, consistent with mRNA changes (Fig. 6, *C* and *D*). In addition, in A549 cells overexpressing FLAG-BAP18 plasmid, BAP18 expression levels were significantly elevated, and CCND1, c-myc, and CD44 protein levels correspondingly increased (Fig. 6, *E* and *F*). These results suggest that BAP18 can influence the transcription and protein levels of downstream target genes in the β-catenin-mediated Wnt signaling pathway.

### BAP18 recruits ACTL6A and PAF1 to the β-catenin-mediated Wnt target gene promoter

**Figure 7:**
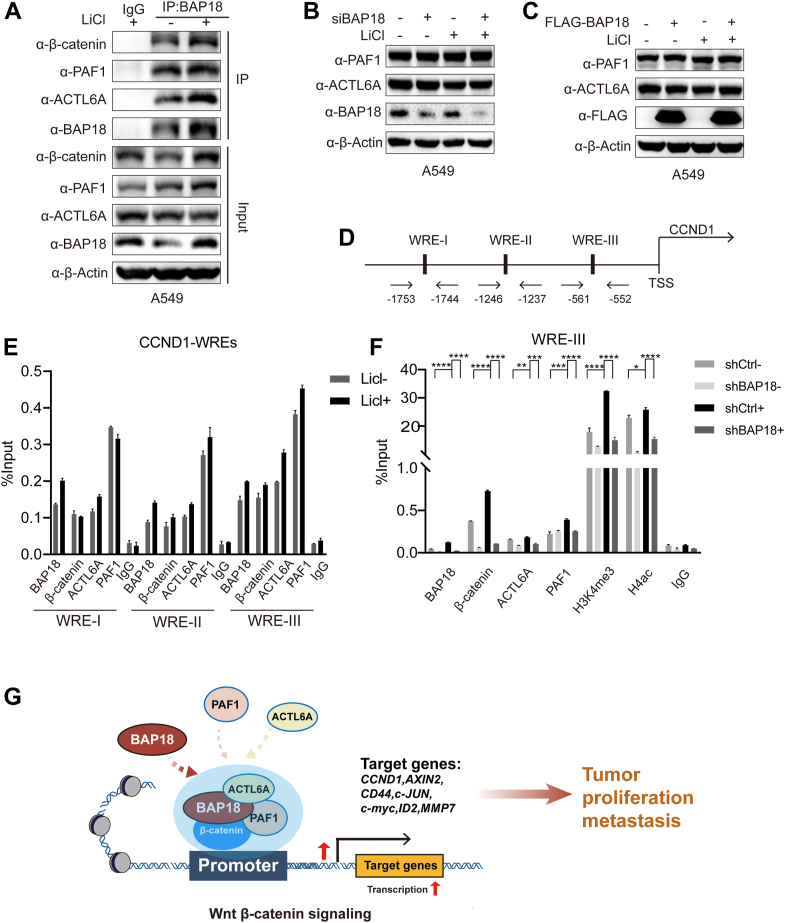
**BAP18 forms a transcriptional complex with β-catenin, ACTL6A, and PAF1 to promote target gene activation at the CCND1 WRE-III region.***A*, co-IP showing endogenous interactions among BAP18, ACTL6A, PAF1, and β-catenin in A549 cells, with or without LiCl stimulation. *B* and *C*, Western blot analysis of ACTL6A and PAF1 protein levels in A549 cells after BAP18 knockdown using siRNA (*B*) or overexpression *via* FLAG-BAP18 transfection (*C*), with or without LiCl stimulation. β-Actin was used as a loading control. *D*, schematic representation of the predicted Wnt-responsive elements (WRE-I–WRE-III) in the human CCND1 promoter region, with transcription start site (TSS) annotated. *E*, ChIP assays showing enrichment of BAP18, β-catenin, ACTL6A, and PAF1 at WRE-I, WRE-II, and WRE-III regions of the CCND1 promoter under LiCl stimulation in A549 cells. ChIP signals were quantified by quantitative PCR (qPCR) and normalized to input DNA. *F*, ChIP assays in stable BAP18 knockdown and control A549 cells, treated with 20 mM LiCl for 12 h. Enrichment of BAP18, β-catenin, ACTL6A, PAF1, and histone modifications (H3K4me3, H4ac) at the WRE-III region was assessed by qPCR. *G*, schematic model of the proposed mechanism. Original blots are provided in Fig. S8. Data are shown as mean ± SEM from three independent replicates. Statistical analysis was performed using unpaired two-tailed Student’s *t* test. ∗∗∗∗*p* < 0.0001; ∗∗∗*p* < 0.001; ∗∗*p* < 0.01; and ∗*p* < 0.05. ACTL6A, actin like 6A; BAP18, BPTF-associated protein of 18 kDa; CCND, cyclin D; ChIP, chromatin immunoprecipitation; Co-IP, coimmunoprecipitation; LiCl, lithium chloride; PAF1, polymerase-associated factor 1; WRE, Wnt responsive element.

Our study confirmed that BAP18 can interact with β-catenin in NSCLC cells, upregulating β-catenin-mediated transcriptional activity and promoting Wnt signaling pathway gene expression. However, as a "reader" of histone H3K4me3, BAP18 requires "writers" or "erasers" to alter chromatin epigenetic signals, thereby recruiting relevant proteins to change chromatin structure and activate gene transcription. To identify potential BAP18-associated coregulators, we performed shotgun LC–MS/MS on immunoprecipitates from cells overexpressing FLAG-tagged BAP18 or empty vector controls. Comparative analysis revealed several candidate interactors enriched in the BAP18 group, among which ACTL6A and PAF1 were prioritized for further investigation based on their detection with unique peptides and known involvement in chromatin remodeling and transcriptional regulation. These findings are detailed in Table 2. Subsequent endogenous coimmunoprecipitation (co-IP) experiments in A549 cells confirmed that BAP18 could interact with β-catenin, ACTL6A, and PAF1 (Fig. 7*A*). We also examined the impact of BAP18 expression on ACTL6A and PAF1. The results indicated that knocking down or overexpressing BAP18 led to a corresponding decrease or increase in BAP18 protein levels, whereas the protein levels of ACTL6A and PAF1 remained largely unchanged (Fig. 7, *B* and *C*).

**Table 2:** Mass spectrometry–based identification of ACTL6A and PAF1 in BAP18-associated protein complexes

Gene name	Coverage/%	Peptides	PSMs	Unique peptides
CTNNB1	18	10	12	8
ACTL6A	3	1	1	1
PAF1	3	1	1	1

Mass spectrometry analysis was performed to identify proteins interacting with BAP18. The table shows representative hits including CTNNB1 (β-catenin), ACTL6A, and PAF1. For each protein, values represent the percentage sequence coverage, total number of peptides identified, peptide-spectrum matches (PSMs), and number of unique peptides. The full list of mass spectrometry–identified proteins is provided in the Supplementary Table.

To determine whether BAP18, β-catenin, ACTL6A, and PAF1 can be recruited to the Wnt downstream gene response element region (WRE) under LiCl stimulation, we conducted chromatin immunoprecipitation (ChIP) assays in A549 cells. CCND1 is a well-known downstream target gene of the Wnt signaling pathway, so we selected it as the target gene for our study. Database predictions identified three potential β-catenin–TCF binding sites (WRE) in the promoter region of CCND1, named CCND1-WRE-I, CCND1-WRE-II, and CCND1-WRE-III, and designed corresponding primers for each site (Fig. 7*D*). ChIP results showed that BAP18, β-catenin, ACTL6A, and PAF1 were mainly recruited to the CCND1-WRE-III region, indicating that β-catenin primarily binds to this region to execute its transcriptional role on target genes (Fig. 7*E*).

To investigate the impact of BAP18 on β-catenin recruitment, we performed ChIP assays in A549 cells with stable BAP18 knockdown (shBAP18) and control cells (shCtrl) under LiCl stimulation, using NaCl as a control. We observed the recruitment levels and histone modifications H3K4me3 and H4ac. The results showed that in the BAP18 knockdown group, the recruitment of β-catenin, ACTL6A, and PAF1 to the CCND1-WRE-III region decreased, accompanied by reduced levels of H3K4me3 and H4ac (Fig. 7*F*).

## Discussion

Lung cancer is the leading cause of cancer-related mortality in men over 40 and women over 60 years (32). Despite stringent public health measures to reduce lung cancer incidence through smoking cessation and early lung cancer screening programs that have decreased mortality risk, a substantial proportion of patients present with advanced-stage disease at diagnosis, precluding curative treatment (33). Historically, chemotherapy has been the mainstay of treatment for advanced lung cancer and continues to play a central role in current clinical practice. Over the past decade, the discovery of predictive biomarkers has enabled the development of targeted therapies and immunotherapies, which are now often used in combination with chemotherapy to enhance treatment efficacy and broaden therapeutic options (34, 35). Although these treatments have significantly improved the prognosis of NSCLC patients, resistance to these treatments inevitably develops (36, 37, 38), necessitating the identification of novel therapeutic targets.

TCGA and the Human Epigenome Project have elucidated the intratumoral and intertumoral heterogeneity, highlighting that even in lung cancers driven by the same oncogenes, phenotypic variations exist among different subclonal states. Epigenetic regulation, including DNA methylation, histone modifications, chromatin remodeling, and noncoding RNAs, plays a crucial role in these subclonal states and can persist throughout tumor progression (39). Proteins that modulate chromatin conformation are categorized into "writers," "erasers," and "readers," each serving distinct functions (40). In lung cancer, overexpression of histone methyltransferases for H3 and H4 is associated with poor prognosis (41). For instance, the demethylase LSD1, overexpressed in NSCLC, promotes lung cancer cell proliferation and invasion (42, 43). "Reader" proteins recognize specific histone marks and recruit coregulators to transcriptionally active regions, thereby modulating gene expression (44).

BAP18, an H3K4me3 reader, is enriched in the NURF–BPTF complex and functions as an important chromatin regulatory cofactor (27). It acts as a coactivator of nuclear hormone receptors such as AR and ER, thereby promoting the progression of prostate and breast cancers by enhancing histone modifications and transcriptional activation at hormone-responsive elements (28, 29, 45). In triple-negative breast cancer (46) and acute myeloid leukemia (47), BAP18 contributes to tumor progression by regulating chromatin accessibility at enhancer regions or insulator elements, maintaining oncogenic transcription programs. In hepatocellular carcinoma, BAP18 promotes metabolic adaptation and therapy resistance by activating lipid metabolism pathways through peroxisome proliferator–activated receptor alpha–mediated transcription (48). Likewise, in oral squamous cell carcinoma, BAP18 promotes cell cycle progression and proliferation by activating CCND1 and CCND2 transcription *via* MLL1 complex recruitment and H3K4me3 enrichment at their promoters (49). In addition, a previous study reported that BAP18 is upregulated in NSCLC tissues and promotes tumor cell proliferation by transcriptionally activating CCND1 and CCND2, suggesting a potential oncogenic role (30). However, the broader regulatory mechanisms of BAP18 in lung cancer have not been fully elucidated. In the present study, we confirmed and extended these findings by demonstrating that BAP18 expression is significantly elevated in NSCLC tissue samples. Although the increase in transcript levels observed in public datasets appears modest, our independently collected clinical samples showed a more pronounced elevation at the protein level, as determined by Western blot and immunohistochemistry. This discrepancy may reflect differences in patient cohorts, sequencing platforms, or sample processing and could also be influenced by post-transcriptional mechanisms, such as enhanced translational efficiency or increased protein stability.

Knockdown of BAP18 in NSCLC cell lines increased the proportion of cells in the G1 phase, decreased the S phase proportion, slowed cell proliferation, and inhibited tumor growth and migration. These cell cycle changes are consistent with previous findings that BAP18 promotes NSCLC cell proliferation by transcriptionally activating CCND1 and CCND2, two key regulators of the G1–S transition (30). In addition, BAP18 downregulation in BALB/c mouse xenograft models significantly inhibited tumor growth, providing *in vivo* evidence that was not addressed in earlier studies. These findings suggest that BAP18 functions as an oncogene in NSCLC. Interestingly, while BAP18 knockdown affected both cell proliferation and migration, the inhibitory effect on migration appeared more prominent. This observation, together with transcriptomic findings highlighting suppression of pathways such as Rap1 and β-catenin signaling, suggests a potential role for BAP18 in modulating tumor cell motility. Consistent with this observation, clinical sample analysis further revealed that high BAP18 expression was significantly associated with lymph node metastasis (N1–N3, *p* = 0.024) and advanced clinical stage (stage II–III, *p* = 0.030). These associations support the notion that BAP18 may contribute to tumor aggressiveness and metastatic progression in NSCLC. Further investigation will be needed to determine whether this migratory phenotype is a primary mechanism by which BAP18 contributes to NSCLC progression.

To explore the pathways through which BAP18 promotes NSCLC progression, we performed transcriptomic sequencing. Changes in BAP18 expression led to alterations in a series of genes. GO enrichment analysis of DEGs revealed involvement in diverse functions and pathways, including protein binding, cellular processes, cell cycle, and biological regulation. We identified a subset of genes significantly regulated by BAP18 involved in cell growth and tumor development. Enrichment analysis indicated these genes promote tumor progression *via* the β-catenin nuclear translocation signaling pathway. The most well-known pathway mediated by β-catenin is the Wnt–β-catenin signaling pathway, which involves β-catenin translocating from the cytoplasm to the nucleus, binding to TCF–LEF transcription factors, activating TCF–LEF transcriptional activity, and regulating downstream target genes (50). Depending on the downstream target genes, it executes various biological functions, such as CCND1 and c-myc, which are related to cell proliferation, differentiation, cell cycle regulation, and tumor growth (51, 52, 53); CD44, which plays a crucial role in stem cell maintenance (54); and MMPs, which are closely associated with tumor invasion and metastasis (55, 56). BAP18 alteration affects Wnt–β-catenin downstream gene expression. Dual-luciferase reporter assays confirmed that BAP18 overexpression upregulates β-catenin-mediated gene transcription activity upon stimulation with LiCl or recombinant Wnt3a protein, whereas BAP18 knockdown decreases this activity. Silencing BAP18 reduced mRNA and protein levels of a series of Wnt–β-catenin signaling pathway target genes, indicating that BAP18 promotes Wnt–β-catenin target gene transcription, thereby mediating lung cancer progression through the Wnt–β-catenin signaling pathway.

The Wnt signaling pathway is a conserved evolutionary pathway playing a vital role in embryonic development, adult stem cell homeostasis, and tissue regeneration (57). It includes the canonical β-catenin-mediated pathway and noncanonical pathways like Wnt/Ca^2+^ and planar cell polarity pathways (58). The canonical Wnt signaling pathway primarily controls cell proliferation, and its dysregulation is associated with various diseases, including skeletal disorders, pigmentary abnormalities, neurodegenerative diseases, and chronic obstructive pulmonary disease (59, 60, 61, 62). The Wnt–β-catenin pathway plays an oncogenic role in various cancers (63, 64, 65, 66, 67). The Wnt–β-catenin pathway involves extracellular signals, membrane fragments, cytoplasmic fragments, and nuclear fragments. Extracellular signals are mediated by 19 Wnt proteins, including Wnt3a, Wnt1, and Wnt5a (68). The membrane segment contains Wnt receptors Frizzled and LRP5/6. The cytoplasmic segment includes β-catenin, DVL, GSK-3β, AXIN, APC, and CK1. The nuclear fragment includes β-catenin, TCF–LEF family members, and downstream Wnt–β-catenin target genes, such as MMPs and c-Myc. In the absence of Wnt stimulation, the "destruction complex" phosphorylates and ubiquitinates β-catenin, leading to its degradation and inhibiting Wnt target gene transcription. When Wnt signaling is recognized, the "destruction complex" is recruited to the cell membrane, releasing β-catenin into the cytoplasm to enter the nucleus, where it binds to TCF–LEF, promoting gene expression (69).

Wnt–β-catenin pathway dysregulation is often caused by mutations in various pathway components, particularly mutations or silencing of Wnt tumor suppressor genes, Axin1/2 mutations, and GSK3β loss (70, 71, 72). APC mutations are also found in 80% of colorectal adenomas and metastatic colorectal cancers and are among the earliest mutations in colorectal cancer development (73, 74). Unlike the frequent APC mutations in colorectal cancer, APC mutations are rare in lung cancer (75). Gene expression regulation is an extremely complex and precise process, with transcriptional regulation being a major mode of gene control. Recent studies have found that transcriptional regulation requires numerous coregulators, and aberrant expression of coregulators is a significant factor in the Wnt–β-catenin signaling pathway–mediated gene transcription abnormalities. Activation of Wnt target genes does not occur immediately; once activating transcription factors bind to DNA, they need to recruit chromatin remodelers, mediator complexes, and coactivators to alter chromatin structure near gene transcription sites, anchoring and activating RNA polymerase II at the transcription initiation site (76). Over 10 transcriptional coactivators are involved in β-catenin target gene activation, including FHL2, TBP, and CBP/p300 (77), making gene expression regulation more efficient and precise.

To explore the microenvironment of BAP18's upregulation of β-catenin, we used proteomics to identify proteins cobinding with BAP18. ACTL6A, formerly known as BAF53A, is part of the SWI–SNF chromatin remodeling complex, altering chromatin structure by changing DNA–histone contacts within nucleosomes in an ATP-dependent manner (78). It is also a component of the NuA4 histone acetyltransferase complex, primarily participating in transcriptional activation by acetylating nucleosomal histone H4 and H2A (79). Recent studies show that ACTL6A is involved in β-catenin-mediated gene expression regulation, impacting transcriptional regulation, DNA double-strand repair, and nuclear factor aggregation. PAF1 is a component of the PAF1 complex (PAF1C), crucial for the development and maintenance of embryonic stem cell pluripotency (80). PAF1C is reported to be necessary for the transcription of Hox and Wnt target genes (81). PAF1C participates in histone modifications, such as histone H2B ubiquitination and H3K4me3 methylation, thereby regulating gene transcription (82). Histone H3K4me3 and H4ac are closely associated with transcriptional activity, and experiments have confirmed that BAP18 can interact with both. BAP18, β-catenin, ACTL6A, and PAF1 can be recruited to the promoter region of the classical Wnt target gene CCND1. Silencing BAP18 reduces the recruitment levels of β-catenin, ACTL6A, and PAF1, accompanied by decreased recruitment of histone modifications H3K4me3 and H4ac, demonstrating that BAP18 interacts with ACTL6A and PAF1 to synergistically upregulate β-catenin transcription. Thus, we hypothesize that BAP18, ACTL6A, and PAF1 form a complex, enhancing chromatin accessibility at target gene promoter regions or other regulatory regions, which may require further assay for transposase-accessible chromatin using sequencing to elucidate chromosomal complexity (45).

## Conclusion

To sum up, in NSCLC, BAP18 is recruited to the promoter region of Wnt target genes, where it facilitates the recruitment of β-catenin, ACTL6A, and PAF1, and enhances the levels of histone modifications H3K4me3 and H4ac, promoting transcriptional activation of Wnt–β-catenin target genes and enhancing lung cancer cell proliferation and migration (Fig. 7*G*). This indicates that BAP18 could serve as a potential therapeutic target for NSCLC.

## Experimental procedures

### Human ethics approval

All human lung tissues were obtained from the First Hospital of China Medical University. All experiments involving human samples were conducted in accordance with the relevant guidelines and regulations and were approved by the Ethics Committee of the First Hospital of China Medical University. Informed consent was obtained from all participants and/or their legal guardians. The human studies reported in this article abide by the principles of the Declaration of Helsinki.

### Plasmids and antibodies

Expression plasmids for FLAG-β-catenin and pcDNA3.1 were kindly provided by the Chromosome Biology Laboratory at China Medical University. The plasmids for FLAG-BAP18 and GFP-BAP18 were constructed in our laboratory. The antibodies used in this study included anti-BAP18 (Bethyl Laboratories, Sigma), anti-H3K4me3 (Millipore), anti-H4ac (Millipore), anti-β-actin (Proteintech), anti-β-catenin (Cell Signaling and Proteintech), anti-cyclin D1 (Proteintech), anti-GFP tag (Proteintech), anti-c-myc (Proteintech), anti-CD44 (Proteintech), anti-ACTL6A (Proteintech), anti-PAF1 (Proteintech), anti-FLAG tag (GNI), anti-rabbit/mouse IgG (ABclonal), and anti-IgG (Santa Cruz). Antibody specificity was confirmed by observing single bands at expected molecular weights and by comparing expression levels between knockdown and control samples. For selected antibodies, specificity was further validated by peptide competition assays or using siRNA-mediated knockdown of the target protein.

### siRNA and lentivirus

siRNA duplexes targeting BAP18 and control siRNA were purchased from Sigma–Aldrich. The sequences for the siRNA were as follows: siBAP18#1: 5′-GGGACGAUCUUAAUCACAUTT-3′ (antisense: 5′-AUGUGAUUAAGAUCGUCCCTT-3′); siBAP18#2: 5′-CAAGGUAUAUGAAGAUUCUTT-3′ (antisense: 5′-AGAAUCUUCAUAUACCUUGTT-3′); and siBAP18#3: 5′-CCAGCUAAGAAACUCAACUTT-3′ (antisense: 5′-AGUUGAGUUUCUUAGCUGGTT-3′); and siCtrl: 5′-UUCUCCGAACGUGUCACGUTT-3′ (antisense: 5′-ACGUGACACGUUCGGAGAATT-3′). Lentiviral productions for shBAP18 and shCtrl were purchased from GeneChem, with the shBAP18 target sequence being 5′-GGGACGAUCUUAAUCACAUTT-3′ (antisense: 5′-AUGUGAUUAAGAUCGUCCCTT-3′).

### Cell culture and transfections

All cells were cultured at 37 °C in a humidified atmosphere containing 5% CO_2_. A549 and NCI-H1299 cell lines were provided by the Developmental Cell Biology Laboratory at China Medical University, whereas NCI-H1975 cells were purchased from the Shanghai Institute of Cell Biology, Chinese Academy of Sciences. Trans5α chemically competent cells were obtained from TransGen Biotech. The authenticity of all cell lines used in this study was validated by short tandem repeat profiling. Mycoplasma contamination was routinely tested and excluded. A549, NCI-H1299, NCI-H460, and NCI-H1975 cells were grown in RPMI1640 medium (Gibco) supplemented with 10% fetal bovine serum (Clark). LiCl was added to the medium according to the experimental design before collecting cells.

Plasmid transfections were performed using jetPRIME DNA Transfection Reagent (Polyplus transfection) following the manufacturer's instructions. On the day before transfection, cells were plated to reach 60 to 80% confluence by the next day. The required amount of plasmid was calculated based on the experimental design. The plasmid was added to the transfection buffer, vortexed for 10 s, and briefly centrifuged. The transfection reagent was then added, vortexed, briefly centrifuged, and incubated at room temperature for 10 min. The transfection mixture was evenly added to the cells, and the culture dish was gently shaken. After 4 to 6 h, the medium was changed, and the cells were continued to be cultured until the designated time for cell collection. siRNA transfections were performed using the same reagent and following a similar procedure.

For lentiviral transfections, cells were plated in 6-well plates to reach 30% confluence the next day. The culture medium was changed to antibiotic-free medium before infection. The appropriate amount of lentivirus and transfection enhancer GP/GA (as per GeneChem's instructions) was added to the cells. After 48 h, the medium was changed to regular culture medium, and puromycin (1:1000) was added for selection.

### Protein extraction and Western blotting

Cells were lysed in TNE buffer (50 mM Tris–HCl [pH 7.5], 150 mM NaCl, and 0.5% Nonidet P-40) containing protease and phosphatase inhibitors (Roche) by sonication on ice. For protein extraction, cells were washed with PBS and scraped into a 1.5 ml EP tube. The cells were centrifuged at 800 rpm, 4 °C for 5 min, and the supernatant was discarded. TNE buffer with protease inhibitors was added, and the cells were sonicated. The mixture was then centrifuged at 12,000 rpm, 4 °C for 20 min, and the supernatant was collected as the protein extract.

For Western blotting, protein samples were prepared and loaded onto SDS-PAGE gels. After electrophoresis, proteins were transferred to polyvinylidene fluoride membranes. The membrane was blocked with 5% milk and incubated with primary antibodies overnight at 4 °C. The next day, the membrane was washed and incubated with secondary antibodies for 1 h at room temperature. Bands were visualized using ECL detection reagents. Western blot signals were quantified using ImageJ software (National Institutes of Health, Bethesda, MD). Band intensities were normalized to the loading control. All quantifications were performed from at least three independent experiments, and results were presented as mean ± SD.

### Luciferase assay

In the luciferase assay, cells were plated in 24-well plates and transfected with luciferase reporter plasmids and experimental plasmids according to the manufacturer's instructions. After 24 h, cells were lysed using luciferase lysis buffer. Luciferase activity was measured using a dual-luciferase reporter assay system, normalizing Firefly luciferase activity to Renilla luciferase control activity.

### RNA isolation and quantitative real-time PCR

Total RNA was extracted from cells using Trizol reagent according to the manufacturer’s instructions. RNA concentration was measured, and 1 μg of RNA was reverse transcribed into complementary DNA using the PrimeScript RT–PCR Kit (TAKARA). Real-time PCR assays were performed using ChamQ Universal SYBR qPCR Master Mix (Vazyme) on a LightCycler96 system (Roche). Specific primers for target genes were used, and GAPDH was used as the internal control.

### Immunohistochemistry

Tissue samples were fixed in formalin, embedded in paraffin, and sectioned into 4 μm thick slices. The sections were deparaffinized, rehydrated, and endogenous peroxidase activity was blocked. Antigen retrieval was performed, and the sections were incubated with primary antibodies overnight at 4 °C. The next day, the sections were incubated with biotinylated secondary antibodies, followed by staining with 3,3′-diaminodbenzidine substrate. The sections were counterstained with hematoxylin, dehydrated, and mounted for examination. The immunohistochemistry staining was performed on an LUAD tissue microarray (Shanghai Outdo Biotech Company; catalog no.: HLugA180Su11), and the evaluation of stained slides was performed blindly without prior knowledge of clinical information.

### ChIP assay

ChIP assays were conducted following the standard protocols from Nature Protocols. Cells were fixed with 1% formaldehyde for crosslinking, harvested in lysis buffer, and sonicated on ice. IP of the chromatin solution was performed using antibodies against BAP18, ACTL6A, PAF1, β-catenin, H3K4me3 and H4ac. Protein A-Sepharose beads were used for the IP reaction, and the complexes were washed sequentially with low salt buffer, high salt buffer, LiCl buffer, and TE buffer. The protein–DNA complexes were eluted, reverse-crosslinked, and purified. DNA was dissolved in TE buffer and used as a template for qPCR. Primers used for qPCR were as follows: CCND1-WRE-I: 5′-CGGGCCCCATAAATCATCCA-3′ and 5′-CATAGCCAAGCCTCAGAGCA-3′; CCND1-WRE-II: 5′-TGCACCAAAGAGACAGAACCT-3′ and 5′-CGTGGTTACATGAGAGGGTCC-3′; CCND1-WRE-III: 5′-CGCCGGAATGAAACTTGCAC-3′ and 5′-CGGACAGACGGCCAAAGAAT-3′.

### Cell proliferation, colony formation, scratch assay, and transwell assays

Cell proliferation was measured using the methylthiazol tetrazolium salt assay. Cells were seeded in 96-well plates, and cell viability was measured at specific time points by recording absorbance at 490 nm. For the colony formation assay, cells were cultured in a designated medium, fixed with 4% paraformaldehyde, and stained with Coomassie blue dye. In the scratch assay, lines were uniformly drawn on the back of 6-well plates, and digested cells from the BAP18 knockdown and control groups were seeded into the plates. Once the cells reached confluence, lines were scratched perpendicular to the drawn lines with a pipette tip, washed, and fresh medium was added. Observations and photographs were taken every 6 h. In the transwell assay, migrated cells were fixed with 95% ethanol and stained with 0.1% crystal violet. Images were captured under a microscope.

### Animal experiments

Animal experiments were performed following the Institutional Animal Care and Use Committee of China Medical University guidelines. A549 cells stably expressing shNC or shBAP18 lentivirus were injected into 4-week-old male BALB/c nude mice. Mice were monitored every 7 days for 4 weeks and then euthanized according to humane treatment policies.

### Animal ethics approval

Experimental procedures in animals were performed in accordance with institutional guidelines for the care and use of laboratory animals. The animal protocol was approved by the China Medical University-Institutional Animal Care and Use Committee (CMU-IACUC: protocol no.: CMU2021315). This study is reported in accordance with ARRIVE (Animal Research: Reporting of In Vivo Experiments) guidelines. At the end of the experimental period, all animals were euthanized under mild anesthesia using ketamine (25 mg/kg) and xylazine (3 mg/kg), administered intraperitoneally, ensuring minimal distress.

### Flow cytometry

BAP18 knockdown cells and control cells were seeded into 6-well plates to reach 50% confluence after 24 h. After 24 h, the medium was replaced with antibiotic-free and serum-free medium, and cells were cultured for another 24 h to arrest them in the G0 phase. The medium was then changed to normal medium, and cells were cultured for another 24 h. Cells were trypsinized, centrifuged at 800 rpm at room temperature for 5 min, and washed once with cold PBS. Cells were resuspended in precooled PBS, centrifuged at 800 rpm at 4 °C for 5 min, and the supernatant was discarded. Cells were fixed overnight at 4 °C with 1 ml of precooled 70% ethanol. The next day, cells were centrifuged, washed once with precooled PBS, resuspended in 300 to 500 μl of propidium iodide, and incubated in the dark at room temperature for 15 min. Samples were analyzed by flow cytometry.

### RNA sequencing

A549 cells were cultured and divided into experimental and control groups. Cells were treated with BAP18 knockdown, preserved in Trizol, and sent to Kangce Technology Co, Ltd for RNA extraction, quantification, quality control, reverse transcription, and sequencing. High-quality data were obtained for bioinformatics analysis, and GO and Kyoto Encylopedia of Genes and Genomes enrichment analyses were performed to identify biological functions and pathways of DEGs.

### Proteomics analysis

Cells for transfection were divided into the empty vector control group and the BAP18 overexpression group (FLAG-BAP18). Cells were plated in 100 mm culture dishes and transfected the next day. After 48 h of transfection, cells were collected. IP was performed using anti-FLAG M2 magnetic beads. The immunoprecipitated beads were partially used for SDS-PAGE, and the remaining beads were frozen at −80 °C and sent to Genechem Co, Ltd for mass spectrometry analysis. For on-bead digestion, beads were washed twice with 50 mM ammonium bicarbonate and incubated with sequencing-grade trypsin (Promega) at 37 °C overnight. Resulting peptides were desalted using C18 stage tips and dried in a vacuum concentrator. Peptides were separated on an EASY-nLC 1200 system (Thermo Fisher) with an Acclaim PepMap RSLC C18 column (75 μm × 25 cm) using a 120 min gradient from 5% to 30% solvent B (0.1% formic acid in acetonitrile) at 300 nl/min, then analyzed on an Orbitrap Fusion Lumos mass spectrometer (Thermo Fisher) operating in data-dependent acquisition mode. MS1 scans were acquired at 120,000 resolution (*m/z* 350–1500), automatic gain control target 4e5, maximum injection time 50 ms; MS2 scans were in higher-energy collision dissociation mode at 30% collision energy, resolution 15,000, automatic gain control target 5e4, maximum injection time 100 ms, and dynamic exclusion 30 s.

### Data download and analysis

LUAD mRNA expression profile data and clinical information were downloaded from TCGA database (https://portal.gdc.cancer.gov/). BAP18 expression was analyzed using the UALCAN online website (http://ualcan.path.uab.edu/index.html). Single-cell sequencing data of NSCLC were downloaded from the Gene Expression Omnibus (GEO) database (https://www.ncbi.nlm.nih.gov/gds/), quality controlled, and processed. Biological function scores for each cell were calculated using single sample gene set enrichment analysis, and the "GSVA" R package was used for processing.

### Statistical analysis

GraphPad Prism 9.1.1 software (GraphPad Software, Inc) was used for statistical analysis. Data were expressed as mean ± SD. Statistical outliers were identified using Grubbs’test prior to statistical comparisons. Normality of data distribution was assessed using the Shapiro–Wilk test before applying Student’s *t* test. Student’s *t* test was used to determine significant differences, with *p* < 0.05 considered statistically significant (∗∗∗∗*p* < 0.0001; ∗∗∗*p* < 0.001; ∗∗*p* < 0.01; ∗*p* < 0.05; ns, no statistical significance).

## Data availability

Public database information used in this study was obtained from the TCGA (https://portal.gdc.cancer.gov/) and the GEO (https://www.ncbi.nlm.nih.gov/geo/). The RNA-sequencing data generated and analyzed in this study have been deposited in the GEO under accession number GSE299634. Detailed proteomics results, including identified proteins, peptides, and associated quantification metrics, are provided in the Supporting information (Table). The remaining datasets generated and/or analyzed during the current study are available from the corresponding author upon reasonable request.

## Supporting information

This article contains Supporting information.

## Conflict of interest

The authors declare that they have no conflicts of interest with the contents of this article.
