# Supplementary material for: The chromatin remodeling factor BAP18 promotes non–small cell lung cancer progression via the recruitment of β-catenin with the transcriptional coactivator complex ACTL6A–PAF1
Source: J Biol Chem. 2025 Aug 14;301(9):110596. doi: 10.1016/j.jbc.2025.110596 (PMC12455121; doi:10.1016/j.jbc.2025.110596)
Supplement: Supporting Information [file mmc2.docx]

**Supporting Information**

**The chromatin remodeling factor BAP18 promotes non-small cell lung cancer progression via the recruitment of β-Catenin with the transcriptional coactivator complex ACTL6A-PAF1**

Junli Hao^1#^, Qilin Hu^1#^, Xin Li^1#^, Sha Shi^1#^, Fangjian Na^2^, Kai Zeng^3^, Hao Li^3^, Yue Zhao^3*^, Mingfang Zhao^1*^

^1^Department of Medical Oncology, The First Hospital of China Medical University, No. 155 Nanjing North Street, Shenyang, Liaoning province, China.

^2^Network Information Center, China Medical University, 110122, Shenyang, Liaoning province, China.

^3^Department of Cell Biology, Key Laboratory of Medical Cell Biology, Ministry of Education, School of Life Sciences, China Medical University, 110122, Shenyang City, Liaoning Province, China.

^#^ These authors contributed equally to this work

* To whom correspondence should be addressed

**
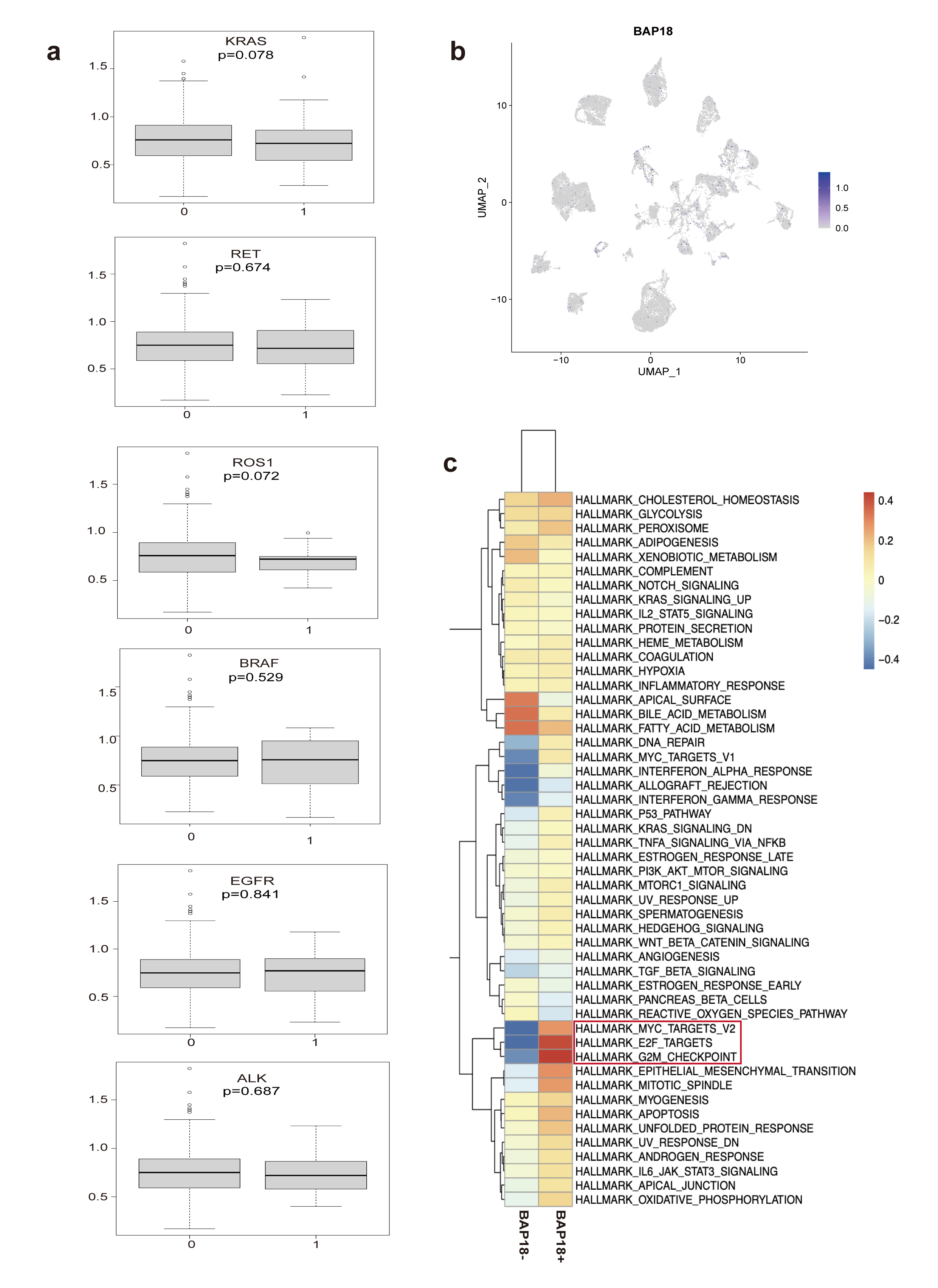
**

**Supplementary Figure 1. Bioinformatics analysis of BAP18 expression and associated pathways.** a. Box plots showing the distribution of BAP18 expression in NSCLC patient samples grouped by mutation status of six common driver genes: KRAS, RET, ROS1, BRAF, EGFR, and ALK. Mutation status is indicated as 0 (wild-type) or 1 (mutant). P-values were calculated using the Wilcoxon rank-sum test. b. UMAP plot showing the distribution and expression intensity of BAP18 in individual cells from the GSE131907 single-cell RNA-seq dataset. Blue represents high expression. c. Single-sample gene set enrichment analysis (ssGSEA) based on hallmark gene sets showing pathways enriched in BAP18-positive (BAP18⁺) versus BAP18-negative (BAP18⁻) cells. Enrichment scores are visualized as a heatmap, with pathways hierarchically clustered.

**
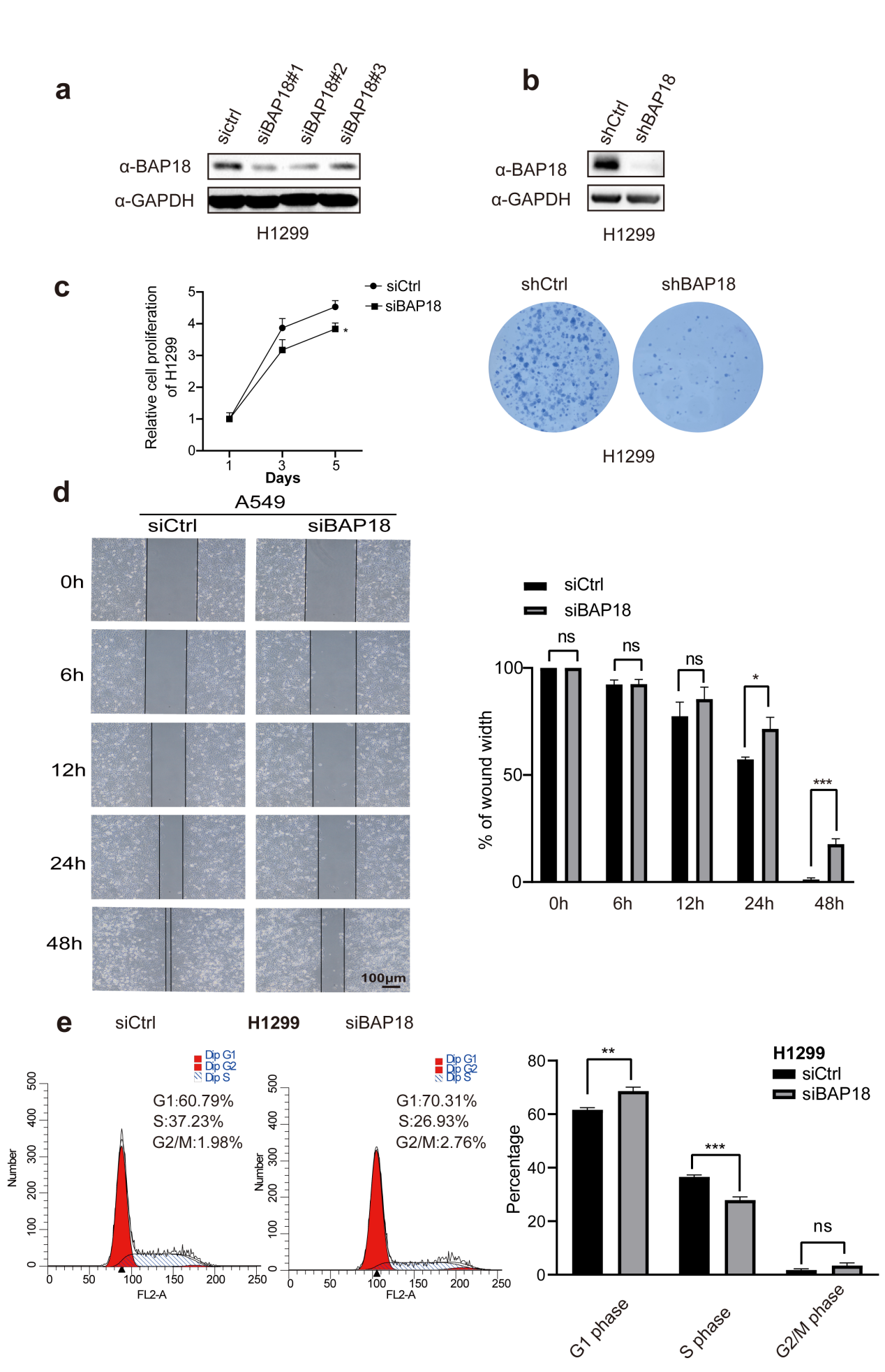
**

**Supplementary Figure 2. BAP18 knockdown suppresses proliferation, migration, and cell cycle progression in NSCLC cells.** a. Western blot analysis confirming BAP18 knockdown efficiency by three independent siRNAs in H1299 cells. GAPDH was used as a loading control. b. Validation of BAP18 knockdown in H1299 cells using lentiviral shRNA constructs. c. Cell proliferation was measured over 5 days by CCK-8 assay in A549 cells transfected with siCtrl or siBAP18. Colony formation assays were performed in H1299 cells with stable knockdown. d. Wound healing assays showing the migration ability of A549 cells following siBAP18 or siCtrl treatment. Representative images were taken at 0, 6, 12, 24, and 48 h. Quantification of wound closure (%) is shown on the right. e. Flow cytometry analysis of cell cycle distribution in H1299 cells after BAP18 knockdown. Representative histograms are shown (left), and phase-specific quantification (right) indicates an increased proportion of G1 phase and decreased S phase cells upon BAP18 depletion. Original blots are presented in Supplementary Figure 9. Data represent mean ± SEM from three independent experiments. Statistical analysis was performed using unpaired two-tailed Student’s t-test. ****P<0.0001; ***P<0.001; **P<0.01; *P<0.05; ns, no statistical significance.

**Supplementary Figure 3: Original Western blot blots of Figure 1c.**

Western blots were cropped prior to incubation with primary antibody hybridization.


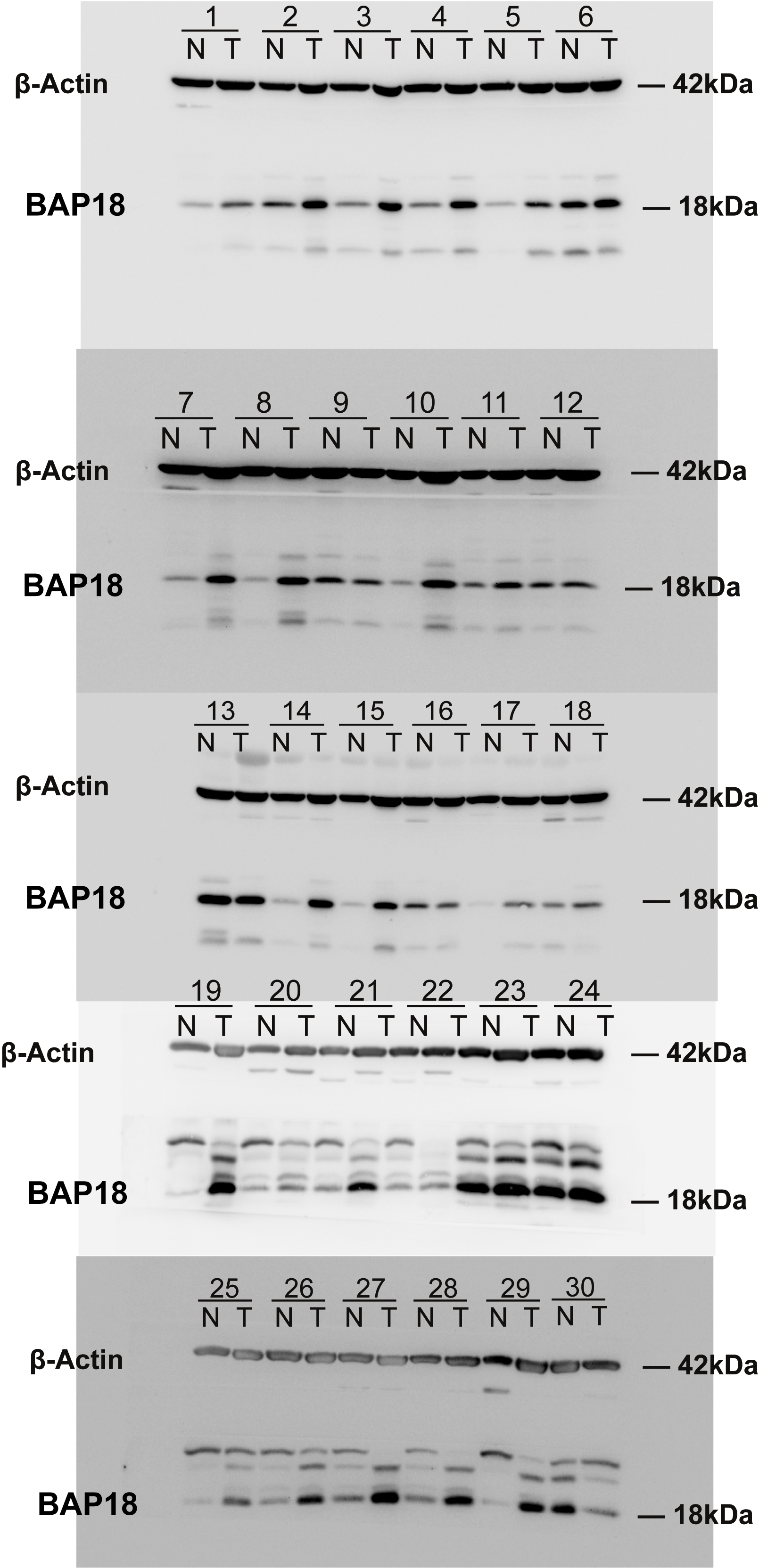


**Supplementary Figure 4:** **Original Western blot blots of Figure 2a-c.**

Western blots were cropped prior to incubation with primary antibody hybridization.


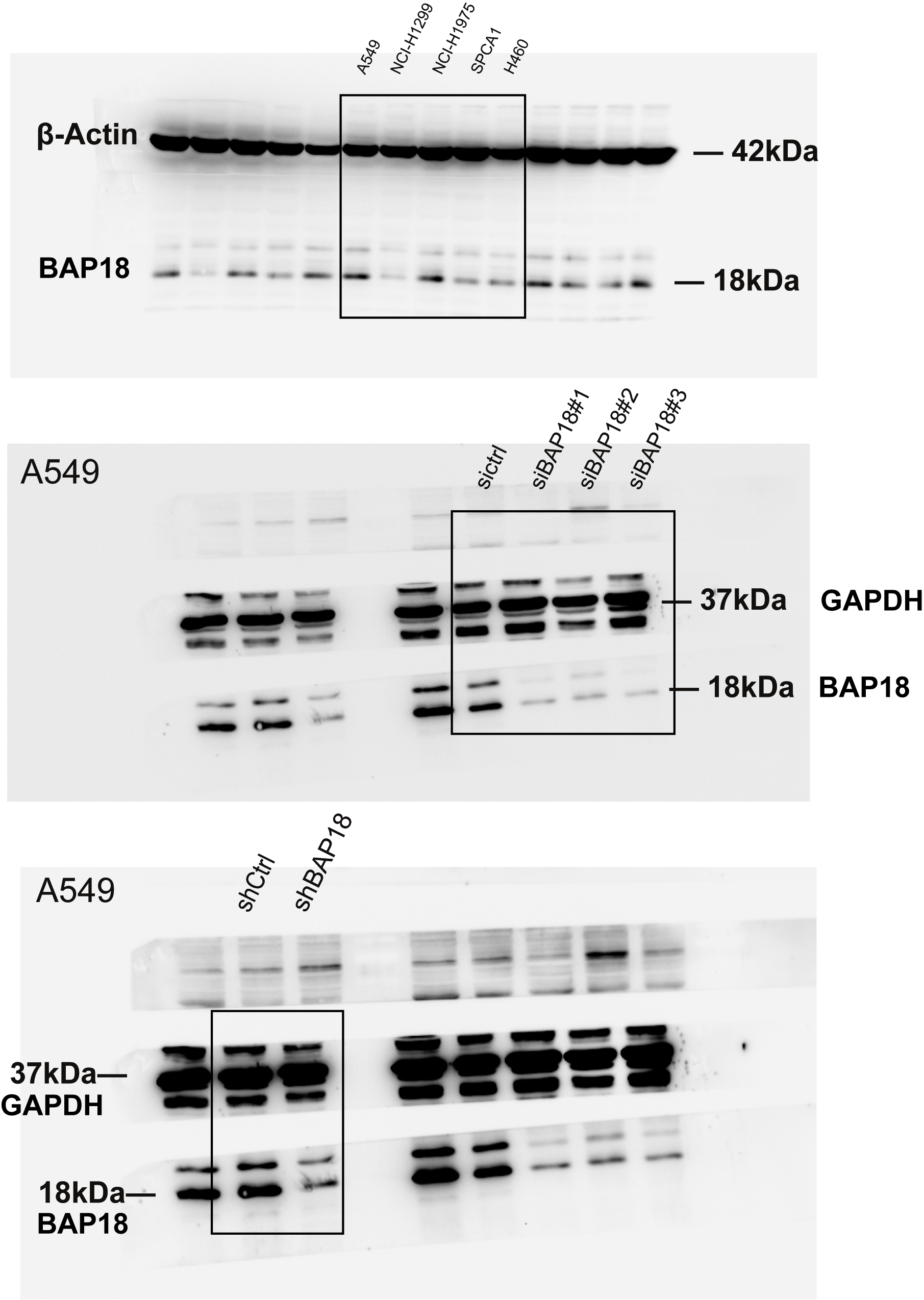


**Supplementary Figure 5: Original Western blot blots of Figure 3e.**

Western blots were cropped prior to incubation with primary antibody hybridization.


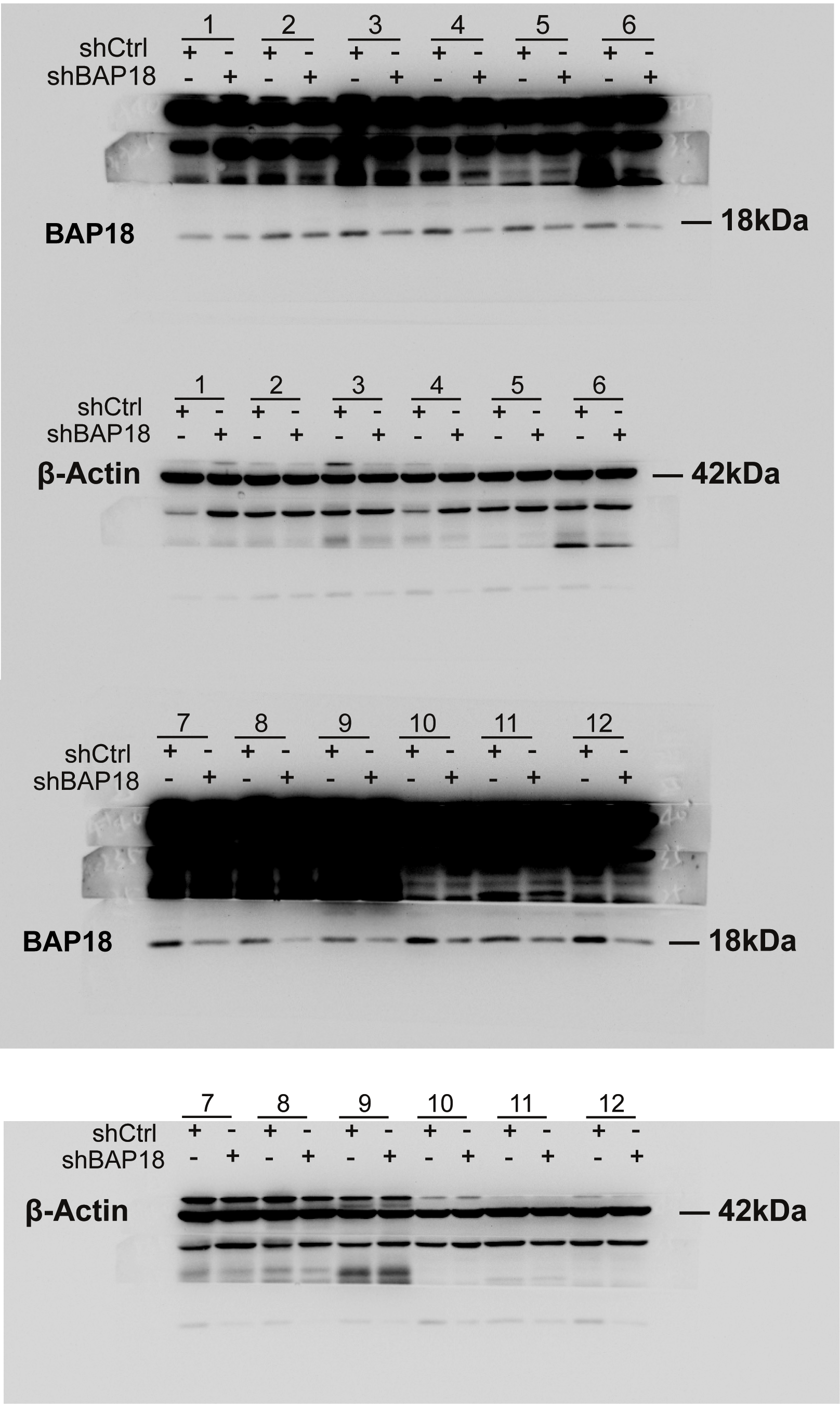


**Supplementary Figure 6: Original Western blot blots of Figure 5.**

Western blots were cropped prior to incubation with primary antibody hybridization.


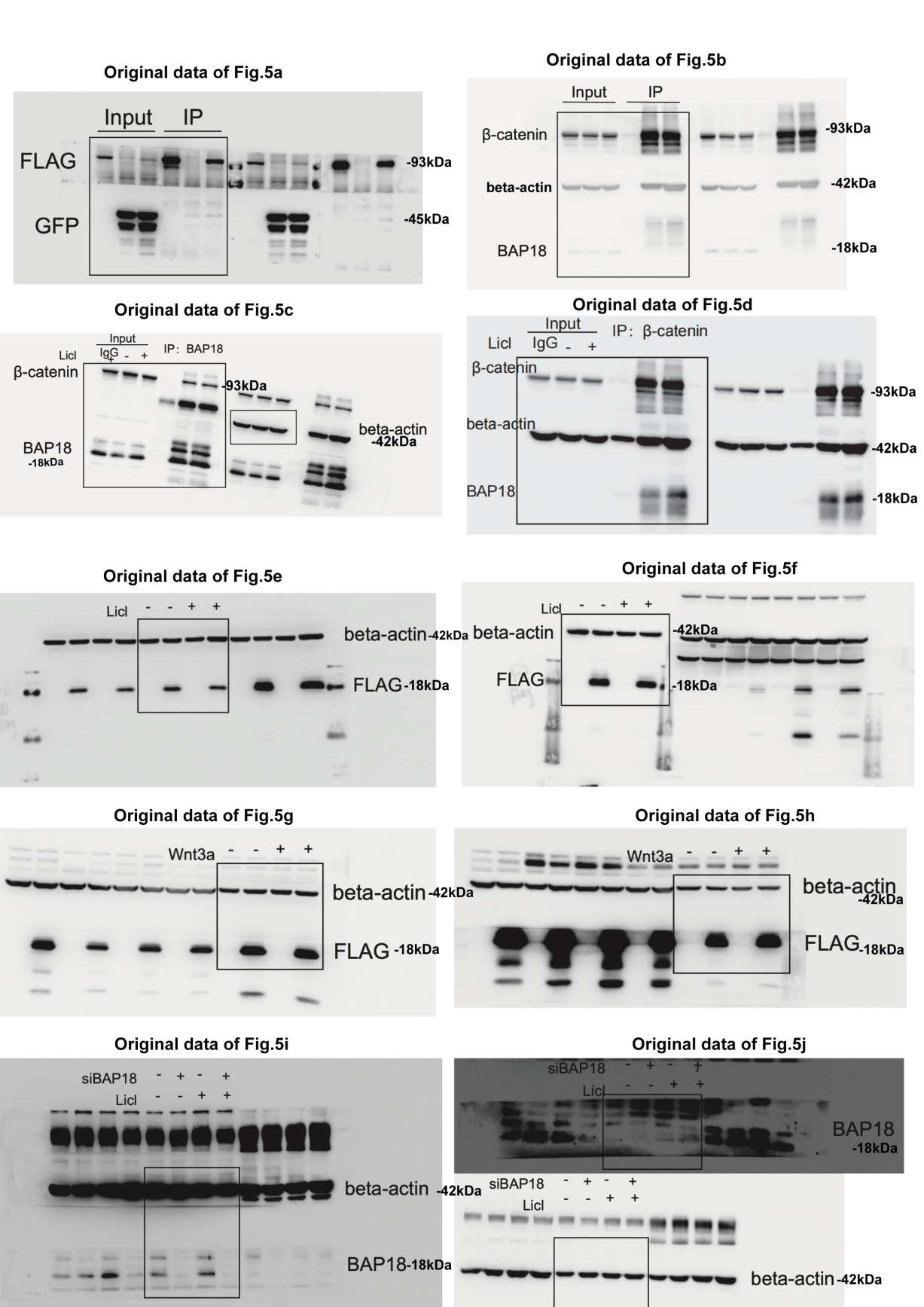


**Supplementary Figure 7: Original Western blot blots of Figure 6c-f.**

Western blots were cropped prior to incubation with primary antibody hybridization.


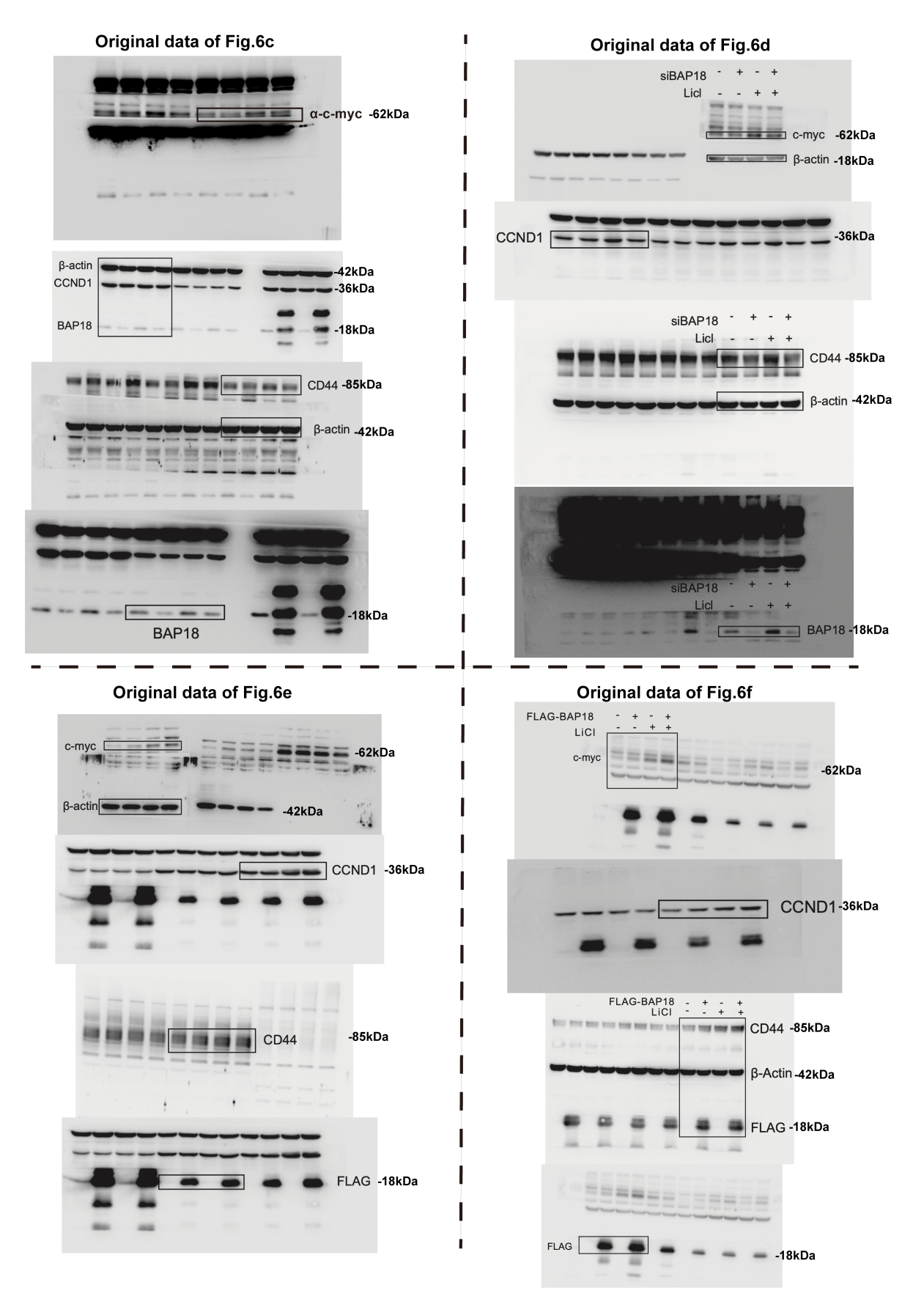


**Supplementary Figure 8: Original Western blot blots of Figure 7a-c.**

Western blots were cropped prior to incubation with primary antibody hybridization.


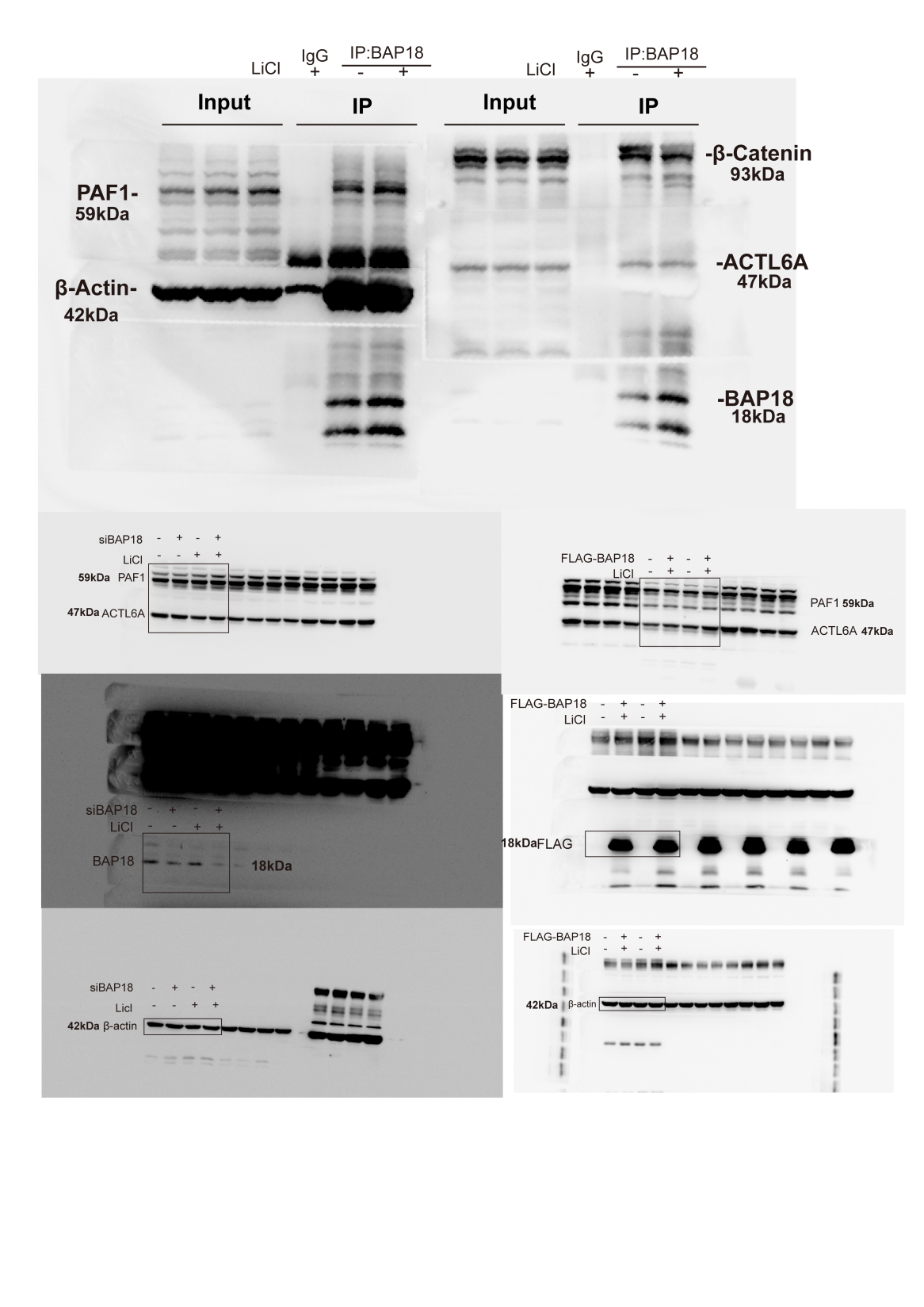


**Supplementary Figure 9: Original Western blot blots of Supplementary Figure 2a-b.**

Western blots were cropped prior to incubation with primary antibody hybridization.

**
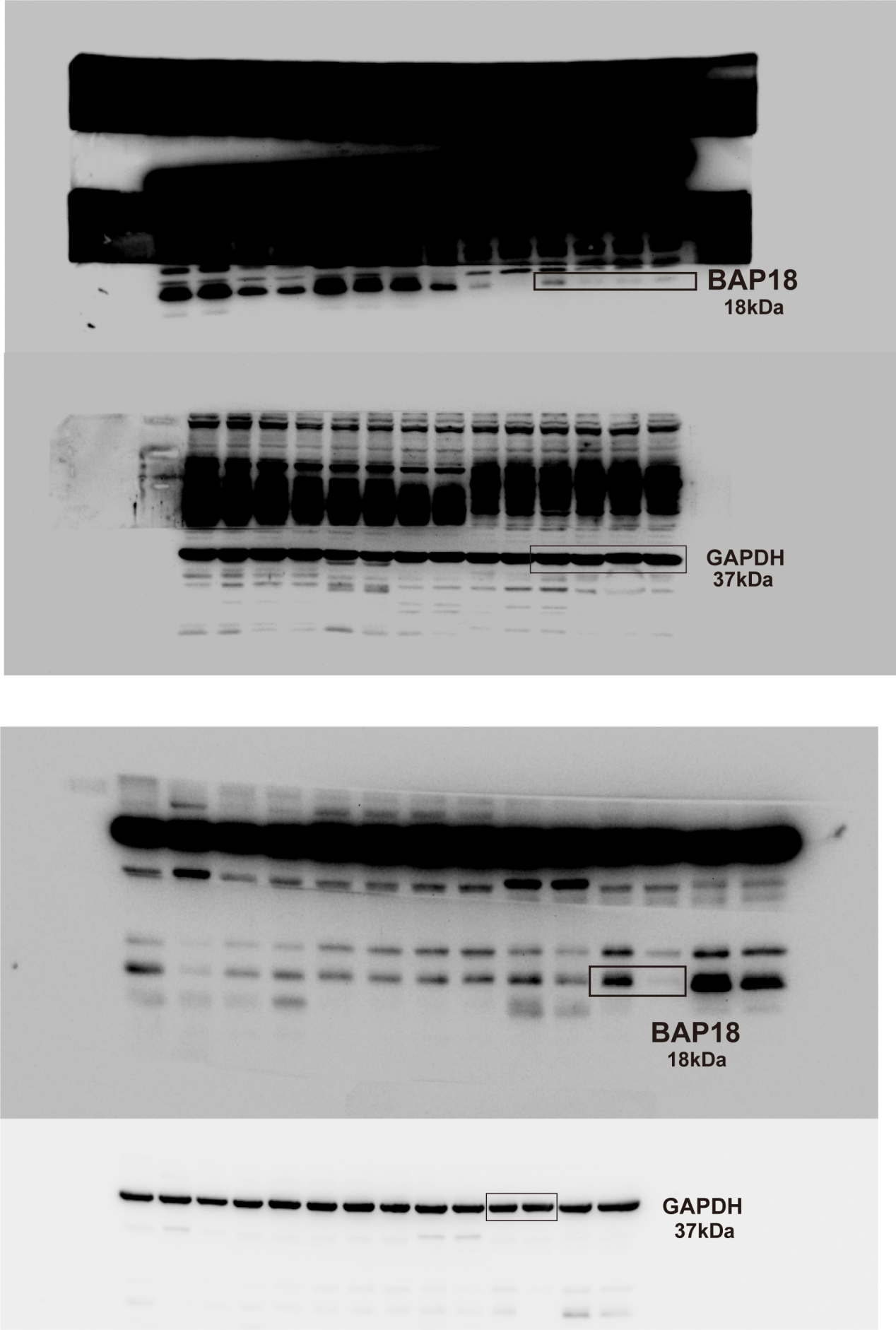
**

**Supplementary Table.** Complete list of proteins and peptides identified by LC-MS/MS following BAP18 co-immunoprecipitation in both control and BAP18-overexpression (OE) groups.
